# Lactylation‐Driven PROS1‐TYRO3‐CARF Signaling Promotes Therapy‐Induced Senescence Escape and Radioresistance in Meningioma

**DOI:** 10.1002/advs.77818

**Published:** 2026-09-16

**Authors:** Zhuohang Wang, Qixiang Zhang, Keman Liao, Xiaoyu Liu, Hongchi Zhang, Yunsheng Chen, Yuxiao Ma, Yikui Liu, Aoqian Xu, Yuhao Sun, Jiayi Chen, Liuguan Bian, Baofeng Wang

**Affiliations:** ^1^ Department of Neurosurgery Ruijin Hospital Shanghai Jiao Tong University School of Medicine Shanghai China; ^2^ Department of Radiation Oncology Ruijin Hospital Shanghai Jiao Tong University School of Medicine Shanghai China; ^3^ Shanghai Key Laboratory of Proton‐Therapy Shanghai China; ^4^ Institute of Radiation Oncology Union Hospital Tongji Medical College Huazhong University of Science and Technology Wuhan China

**Keywords:** CARF, cellular senescence, histone lactylation, meningioma, PROS1, radioresistance, TYRO3

## Abstract

Radiotherapy is a major treatment for meningioma, but radioresistance remains a key obstacle to durable disease control. Here, we show that escape from therapy‐induced senescence (TIS) is a hallmark of radioresistant meningioma cells and identify a novel glycolysis‐H3K27 lactylation‐PROS1‐TYRO3‐CARF axis underlying this phenotype. Using photon‐derived radioresistant meningioma models established from two independent meningioma cell lines, combined with multi‐omics, CUT&Tag, dual‑luciferase reporter assays, immunoprecipitation‐mass spectrometry, and in vivo xenograft analyses, we found that radioresistant cells display enhanced glycolysis, lactate accumulation, and increased H3K27 lactylation. H3K27la promotes PROS1 transcription, leading to PROS1 secretion and TYRO3 activation. Activated TYRO3 in turn promotes phosphorylation of CARF at tyrosine 8 and triggers its proteasomal degradation, relieving CDKN2A/p53‐associated senescence, and permitting cell cycle re‐entry. Disruption of PROS1 signaling or inhibition of CARF phosphorylation restores senescence‐associated phenotypes and enhances radiosensitivity in vivo. Together, these findings establish a metabolic‐epigenetic‐signaling mechanism that couples glycolytic reprogramming to TIS escape in meningioma and highlight the PROS1‐TYRO3‐CARF axis as a potential radiosensitizing target.

## Graphical abstract

A glycolysis‐driven epigenetic switch promotes therapy resistance in meningioma. Increased lactate production enhances histone H3K27 lactylation, which activates PROS1 secretion. Secreted PROS1 engages TYRO3 signaling to degrade the senescence regulator CARF, allowing cancer cells to escape growth arrest. Disrupting this axis restores senescence and overcomes radioresistance, revealing new therapeutic targets. Created with BioRender.com.

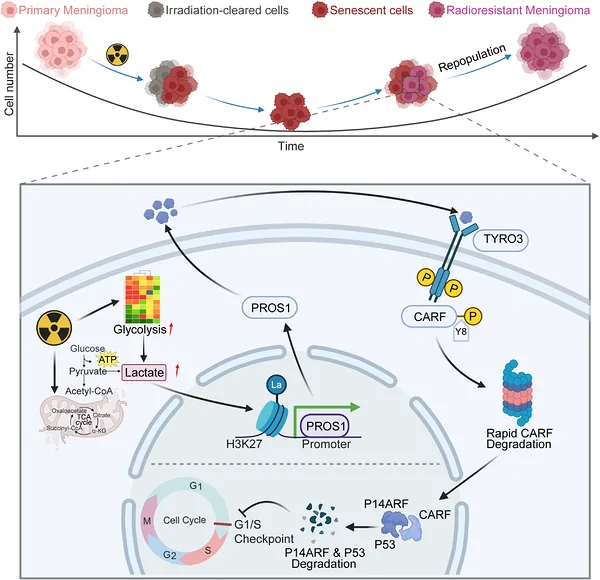

## 1 Introduction

Meningiomas are the most common primary tumors of the central nervous system, accounting for approximately 30%‐40% of all intracranial neoplasms [1, 2]. Although most meningiomas are benign and can be effectively managed by gross‐total resection, approximately 20% of patients present with World Health Organization (WHO) grade 2 or 3 tumors, which are associated with high postoperative recurrence rates and are often difficult to resect completely because of involvement of critical neurovascular structures [3]. In this setting, radiotherapy, particularly photon‐based irradiation, represents a crucial adjuvant treatment for residual, recurrent, or unresectable disease [4]. However, despite its established clinical benefit in prolonging progression‐free survival, a substantial proportion of patients eventually develop local recurrence after irradiation, indicating the presence of intrinsic or acquired radioresistance [5, 6]. Elucidating the molecular basis of meningioma radioresistance is therefore of considerable clinical importance and may facilitate the development of more effective therapeutic strategies.

The antitumor effects of radiotherapy are mediated not only by direct cytotoxicity but also by the induction of therapy‐induced senescence (TIS), a durable state of cell cycle arrest that restricts tumor cell proliferation [7, 8]. In contrast to apoptosis or necrosis, senescence is characterized by distinct phenotypic and molecular features, including increased cell size, the formation of polyploid giant cancer cells (PGCCs), enhanced senescence‐associated β‐galactosidase (SA‐β‐gal) activity, upregulation of cell cycle inhibitors such as p16INK4a and p21CIP1, and the secretion of senescence‐associated secretory phenotype (SASP) factors [9, 10]. TIS was initially regarded as an irreversible endpoint of cancer therapy; however, accumulating evidence now indicates that senescence is not necessarily terminal. A subset of tumor cells can escape from the senescent state after irradiation, re‐enter the cell cycle, and acquire stem‐like or adaptive phenotypes that promote tumor recurrence and therapeutic resistance [11, 12]. Such TIS escape has been reported in multiple tumor types, including lung cancer, breast cancer, and glioblastoma, and has been linked to diverse mechanisms such as p53 dysfunction, Rb pathway inactivation, immune evasion, and SASP‐mediated paracrine signaling [13, 14, 15]. Accordingly, understanding how tumor cells evade TIS has become an important challenge in the effort to improve radiotherapeutic efficacy.

Recent studies have highlighted an important role for epigenetic reprogramming in the regulation of therapy response and senescence‐associated phenotypes [16, 17, 18, 19]. Among these modifications, histone lactylation has emerged as a newly recognized acylation mark that is induced under stress conditions such as hypoxia, senescence, and radiochemotherapy [20, 21]. In tumor cells, enhanced aerobic glycolysis not only supports rapid ATP production and biosynthetic demand but also leads to lactate accumulation within the tumor microenvironment. Once considered merely a metabolic by‐product, lactate was identified by Zhang et al. in 2019 as a direct substrate for histone lysine lactylation, establishing a mechanistic link between metabolism and chromatin regulation [22]. Subsequent studies have implicated histone lactylation, including H3K9la and H3K18la, in processes such as macrophage polarization, immune evasion, chemoresistance, and metastasis [23, 24, 25]. By contrast, histone acetylation, particularly H3K27ac, is generally associated with active chromatin and transcriptional activation, and its reduction has been linked to replicative senescence and DNA damage‐induced senescence [26]. Depletion of H3K27ac at senescence‐associated heterochromatin foci contributes to silencing of cell cycle‐promoting genes, suggesting that loss of H3K27ac may function as an epigenetic constraint on proliferative recovery [27, 28]. These observations raise the possibility that H3K27 lactylation and H3K27 acetylation may exert opposing effects during the senescence response [29]. Defining how glycolysis‐driven epigenetic remodeling shapes radiotherapy‐induced senescence and TIS escape is therefore of substantial biological and translational interest.

PROS1 (protein S) is a vitamin K‐dependent secreted glycoprotein best known for its role as a cofactor for activated protein C in the coagulation cascade [30]. Beyond hemostasis, emerging evidence indicates that PROS1 also functions as a ligand for TAM family receptor tyrosine kinases, including TYRO3, AXL, and MERTK, thereby regulating diverse non‐coagulant processes such as cell survival, apoptosis suppression, inflammatory signaling, and immune microenvironment remodeling [31]. TAM signaling has also been implicated in the regulation of senescence‐related phenotypes [32]. CARF (Collaborator of p14ARF), also known as CDKN2AIP (CDKN2A interacting protein), is a p14ARF interacting factor that stabilizes p14ARF by protecting it from proteasomal degradation [33]. Previous studies have shown that CARF overexpression suppresses proliferation and promotes senescence through the p53‐HDM2‐p21 axis, whereas CARF loss facilitates senescence bypass and malignant transformation [34]. Despite these observations, whether PROS1/TAM signaling participates in TIS escape, and how CARF stability is regulated in this context, remain largely unknown.

Based on these observations, we hypothesized that metabolic reprogramming may enable radioresistant meningioma cells to evade TIS through lactylation‐dependent transcriptional regulation. Specifically, we investigated whether enhanced glycolysis and lactate accumulation promote PROS1 expression through histone H3K27 lactylation, and whether the downstream PROS1‐TYRO3‐CARF signaling axis contributes to TIS escape and radioresistance. To address these questions, we established photon‐radioresistant meningioma cell models from two independent cell lines and integrated transcriptomic, proteomic, metabolomic, epigenomic, biochemical, and in vivo analyses. Our findings identify a mechanistic link between glycolysis‐driven epigenetic remodeling and TIS escape, thereby providing a conceptual framework for targeting meningioma radioresistance.

## 2 Results

### 2.1 Radioresistant Meningioma Cells Exhibit Enhanced TIS Escape Capacity

**FIGURE 1:**
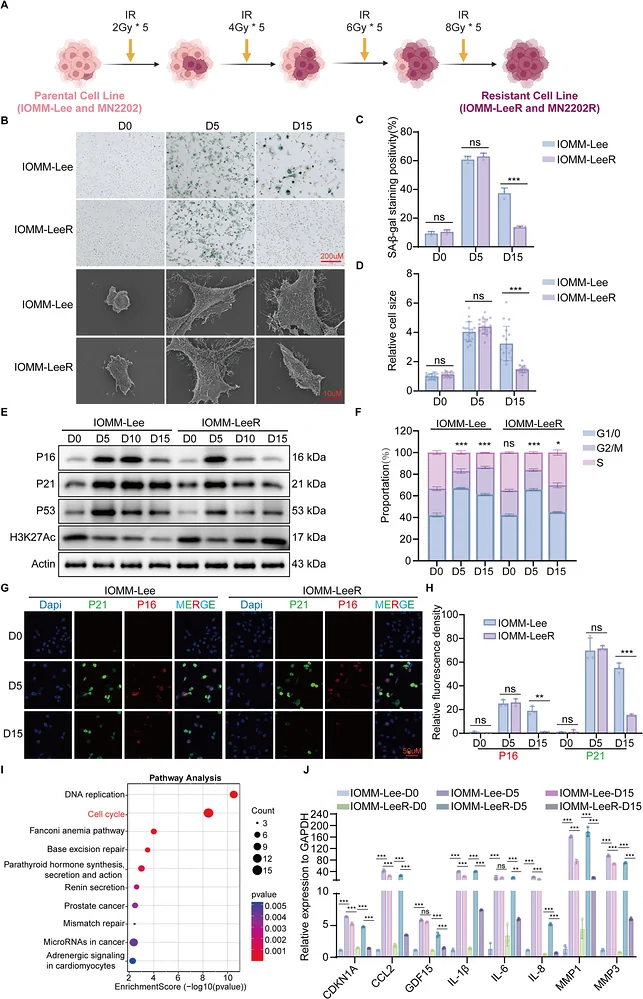
Radioresistant Meningioma Cells Exhibit Enhanced TIS Escape Capacity. (A) Schematic of IOMM‐LeeR and MN2202R generation: cells were subjected to fractionated photon irradiation with escalating doses (2, 4, 6, 8 Gy), each administered 5 times, total cumulative dose 100 Gy. Created with BioRender.com, agreement number: KR2A1FN7PU. (B) Representative SA‐β‐gal staining (top) and scanning electron microscopy images (bottom) of parental (IOMM‐Lee) and resistant (IOMM‐LeeR) cells before irradiation (D0), and at day 5 (D5) and day 15 (D15) post‐8 Gy irradiation. Scale bars: SA‐β‐gal, 200 µm; SEM, 10 µm. (C, D) Quantification of SA‐β‐gal‐positive cell rate (C) (*n* = 3) and relative cell area (D) (*n* = 3, 6 randomly selected cells were measured per individual experiment for area quantification). Unpaired two‐tailed Student's t‐test was applied. (E) Western blotting of senescence markers p16, p21, p53, and H3K27ac protein levels at indicated time points. (F) Flow cytometric cell cycle distribution of both lines at indicated time points (G0/G1 phase compared, *n* = 3). One‐way ANOVA followed by Tukey's multiple‐comparisons test was applied. (G) Immunofluorescence co‐staining of p21 (green) and p16 (red) in both lines at D0, D5, and D15; DAPI (blue). Scale bar: 50 µm. (H) Quantification of p16 and P21 fluorescence intensity (*n* = 3). Unpaired two‐tailed Student's t‐test was applied. (I) KEGG pathway enrichment bubble plot (Top 10) from transcriptomic analysis of IOMM‐Lee vs. IOMM‐LeeR at D15 post‐8 Gy irradiation. (J) qPCR analysis of SASP factors mRNA levels in both lines at D0, D5, and D15 post‐8 Gy irradiation (*n* = 3). One‐way ANOVA followed by Tukey's multiple‐comparisons test was applied. “n” means the number of individual experiments. Data are presented as mean ± SD. *p < 0.05; **p < 0.01; ***p < 0.001; ns, not significant.

We generated radioresistant meningioma cell lines, IOMM‐LeeR and MN2202R, by exposing parental IOMM‐Lee cells to fractionated photon irradiation with escalating doses (2–8 Gy; cumulative dose, 100 Gy; Figure 1A). Colony formation assays showed that IOMM‐LeeR and MN2202R cells had significantly greater survival than parental cells after irradiation, confirming the establishment of a radioresistant phenotype, as demonstrated by representative images across a dose gradient (Figure S1B) and quantitative survival curve analysis for both IOMM‑Lee (Figure S1C) and MN2202 (Figure S1D) pairs. Immunofluorescence further revealed fewer γH2AX foci in resistant cells at 2 h after irradiation, consistent with enhanced DNA damage repair capacity (Figure S1E).

We next compared TIS in the two cell lines. At day 5 after 8 Gy irradiation, both parental and resistant IOMM‐Lee cells displayed typical senescence‐associated features, including increased SA‐β‐gal positivity and enlarged cell size. By day 15, however, IOMM‐LeeR cells showed a marked reduction in SA‐β‐gal staining and cell size, whereas parental cells largely remained in a senescent state (Figure 1B–D). Consistently, western blotting showed that p16, p21, and p53 levels declined from day 5 to day 15 in resistant cells, accompanied by restoration of H3K27ac, whereas parental cells maintained high expression of these senescence‐associated markers (Figure 1E, Figure S4A). Flow cytometry and immunofluorescence further showed that resistant cells re‐entered the cell cycle by day 15, as indicated by release from G0/G1 arrest and increased Ki67 positivity, while parental cells remained arrested (Figure 1F–H; Figure S2A–C). Transcriptomic enrichment analysis identified significant activation of the cell cycle pathway in resistant cells at day 15 (Figure 1I), and qPCR showed dynamic changes in SASP factor expression consistent with these phenotypic shifts (Figure 1J). Consistently, qPCR analysis revealed that CCNA2, CCNB1, and PCNA transcripts, which were suppressed in parental cells at D5 and remained low at D15, showed significant recovery in resistant cells at D15 (Figure S2D). In addition, conditioned media from irradiated parental cells promoted clonogenic survival of unirradiated recipient cells in a dose‐dependent manner (Figure S2E,F), suggesting that SASP components contribute to paracrine survival signals. Collectively, these findings demonstrate that compared with parental cells, radioresistant cells exhibit a markedly enhanced capacity for TIS escape and cell cycle re‐entry after irradiation.

### 2.2 Integrated Multi‐Omics Analyses Reveal Enhanced Glycolysis and Elevated Lactylation in Radioresistant Cells

**FIGURE 2:**
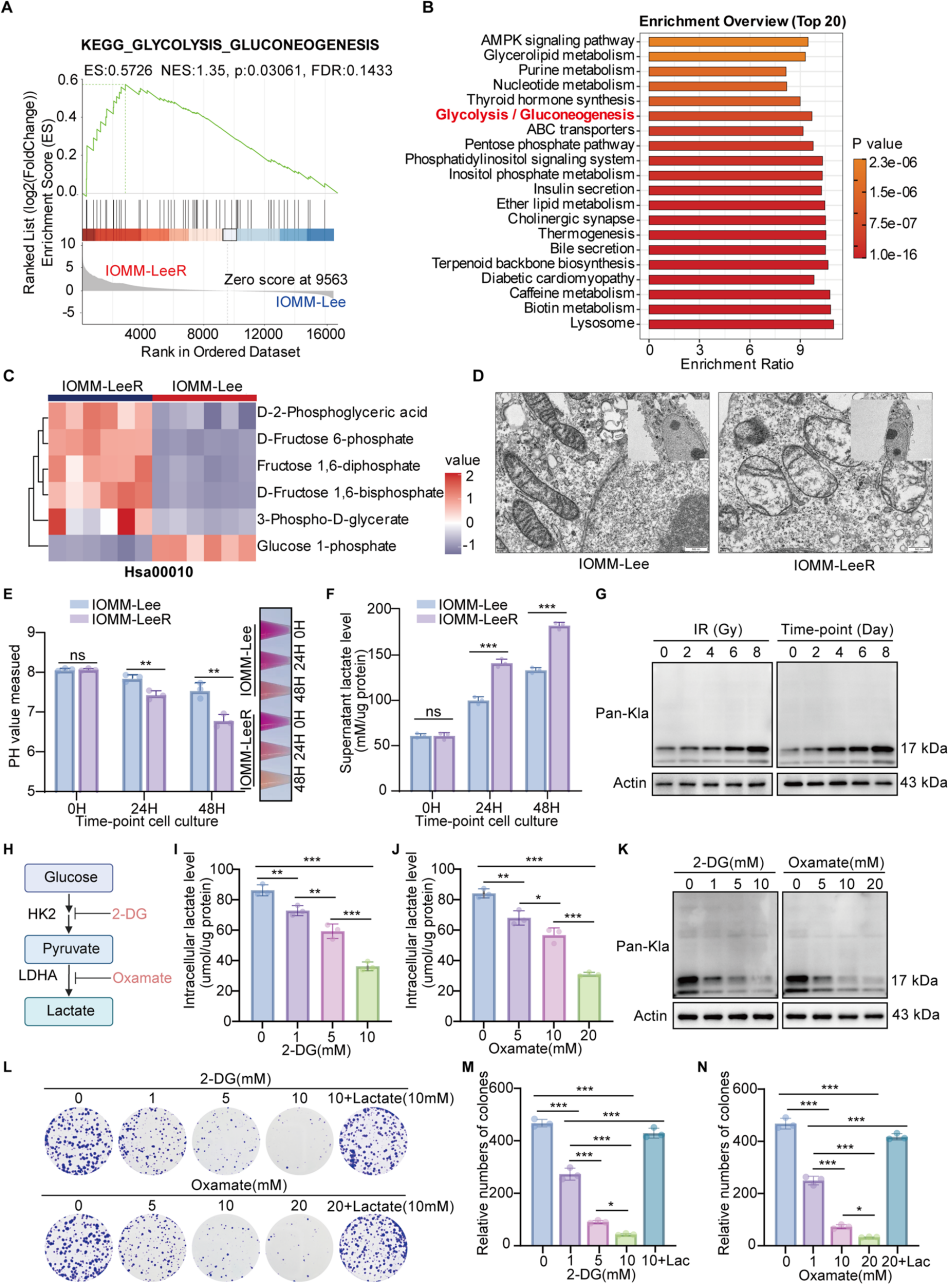
Integrated Multi‐Omics Analyses Reveal Enhanced Glycolysis and Elevated Lactylation in Radioresistant Cells. (A) GSEA enrichment plot showing “glycolysis/gluconeogenesis” significantly enriched in IOMM‐LeeR (NES = 1.35, *p* < 0.05). (B) Metabolomic MSEA enrichment analysis placing “glycolysis/gluconeogenesis” in the top 20 (*p* < 0.01). The glycolysis/gluconeogenesis pathway is highlighted in red. (C) Heatmap of differential fold changes in key glycolytic intermediates from KEGG pathway hsa00010 in IOMM‐Lee vs. IOMM‐LeeR (*n* = 6). (D) Transmission electron microscopy of mitochondrial ultrastructure. Scale bar: 500 nm. (E) Changes in culture supernatant pH and representative medium color of IOMM‐Lee and IOMM‐LeeR cells at 0, 24, and 48 h (*n* = 3). Unpaired two‐tailed Student's t‐test was applied. (F) Lactate levels in culture media of IOMM‐Lee and IOMM‐LeeR cells at 0, 24, and 48 h (*n* = 3). Unpaired two‐tailed Student's t‐test was applied. (G) Western blotting of Pan‐Kla levels in IOMM‐Lee at 24 h post 0, 2, 4, 6, 8 Gy (left) and at 0, 2, 4, 6, 8 days post 8 Gy (right). (H) Schematic of glycolysis inhibitors 2‐DG and Oxamate mechanism. (I, J) Culture media lactate levels in resistant cells treated with increasing concentrations of 2‐DG (I) or Oxamate (J) for 48 h (*n* = 3). One‐way ANOVA followed by Tukey's multiple‐comparisons test was applied. (K) Western blotting of Pan‐Kla levels under the above treatments. (L) Representative colony formation assay of resistant cells treated with 2‐DG, Oxamate, or combined with exogenous lactate after 6 Gy irradiation. (M, N) Quantification of colony formation from (L) (*n* = 3). One‐way ANOVA followed by Tukey's multiple‐comparisons test was applied. “n” means the number of individual experiments. Data are presented as mean ± SD. *p < 0.05; **p < 0.01; ***p < 0.001; ns, not significant.

To define the molecular basis of TIS escape, we performed integrated multi‐omics analyses in parental and resistant cells. Transcriptomic GSEA and metabolomic MSEA consistently showed enrichment of the glycolysis/gluconeogenesis pathway in IOMM‐LeeR cells (Figure 2A,B), untargeted metabolomic profiling revealed a global metabolic shift in radioresistant cells (Figure S3A). KEGG enrichment analysis and DAScore plot analysis identified significant alterations in nucleotide metabolism, central carbon metabolism, as well as pyrimidine and purine metabolism, while glycolysis/gluconeogenesis was consistently ranked as one of the most significantly upregulated pathways across these analyses (Figure S3B,C). Targeted metabolomic analysis further demonstrated increased levels of multiple glycolytic intermediates (Figure 2C, Figure S3D). Transmission electron microscopy revealed swollen and structurally damaged mitochondria in resistant cells, consistent with a metabolic shift toward glycolysis (Figure 2D). Functionally, resistant cells displayed more rapid acidification of the culture supernatant and greater lactate accumulation than parental cells (Figure 2E,F, Figure S3E,F).

Because lactate serves as a substrate for histone lactylation, we next examined lactylation status. Western blotting showed that global lactylation (Pan‐Kla) increased in a time‐dependent manner after 8 Gy irradiation and in a dose‐dependent manner after exposure to 2, 4, 6, and 8 Gy photons (Figure 2G). Inhibition of glycolysis with 2‐DG or Oxamate reduced both lactate levels and global lactylation in resistant cells (Figure 2H–K). Importantly, blockade of glycolysis or lactylation significantly impaired clonogenic survival, indicating a radiosensitizing effect. To exclude nonspecific cytotoxicity of the glycolysis inhibitors, we confirmed that low concentrations of 2‐DG (1 mM) and oxamate (5 mM) did not affect colony formation in the absence of irradiation (Figure S3G). Lactate rescue experiments demonstrated that exogenous sodium lactate supplementation significantly reversed the suppression of clonogenic survival and H3K27la levels caused by 2‐DG or Oxamate, confirming that these effects are mediated through the lactate‐lactylation axis (Figure 2L–N). These findings link radioresistance with enhanced glycolysis, lactate accumulation, and elevated lactylation.

### 2.3 H3K27 Lactylation Promotes PROS1 Expression and Attenuates Senescence‐Associated Phenotypes

**FIGURE 3:**
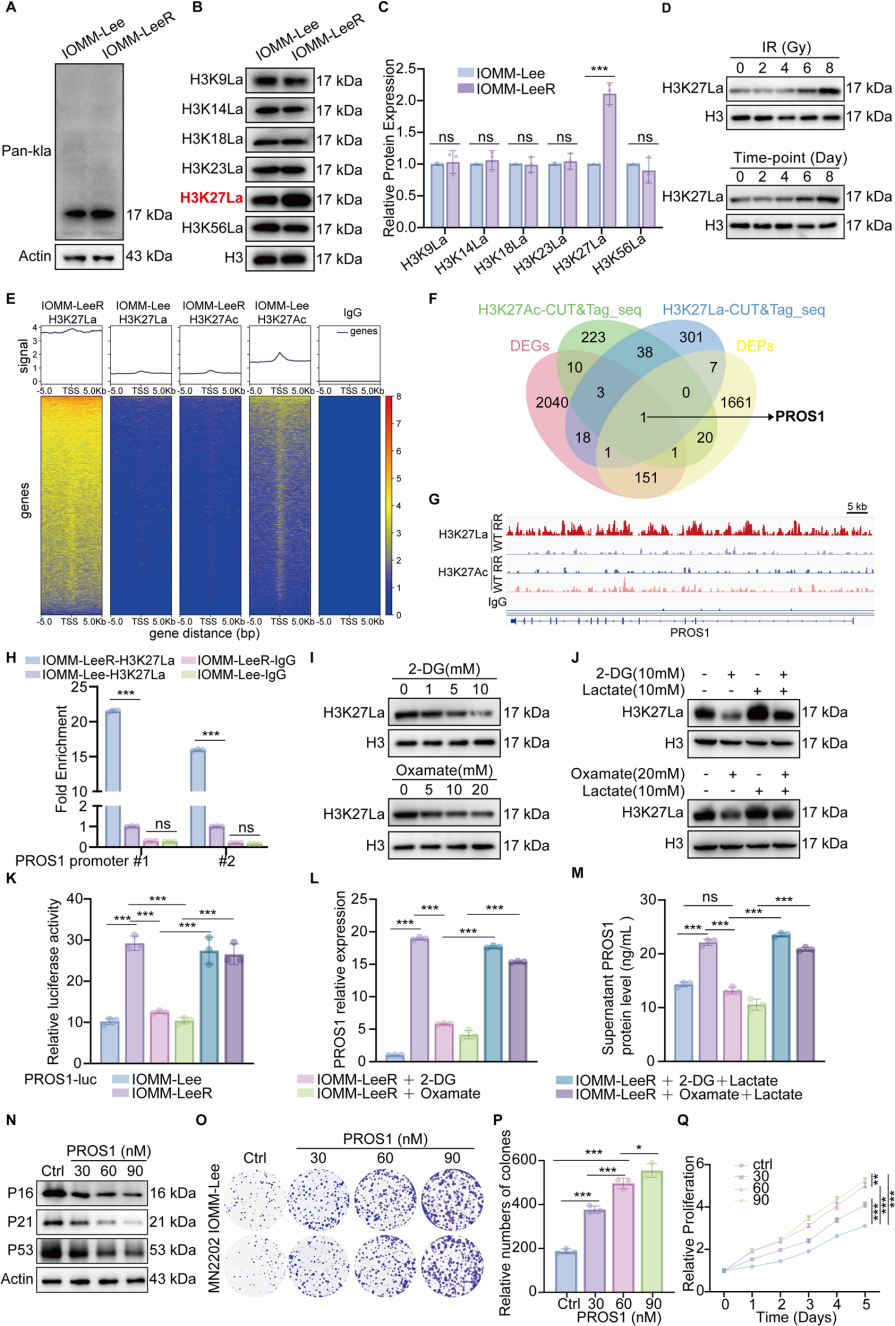
H3K27 Lactylation Promotes PROS1 Expression and Attenuates Senescence‐Associated Phenotypes. (A) Western blotting of Pan‐Kla in IOMM‐Lee and IOMM‐LeeR. (B) Western blot of specific histone H3 lysine lactylation sites (H3K9la, H3K14la, H3K18la, H3K23la, H3K27la, H3K56la) in both lines. (C) Densitometric quantification of each lactylation site normalized to total H3 (*n* = 3). Unpaired two‐tailed Student's t‐test was applied. (D) Western blotting of H3K27la levels in IOMM‐Lee at 24 h post 0, 2, 4, 6, 8 Gy and at 0, 2, 4, 6, 8 days post 8 Gy. (E) Heatmaps of CUT&Tag signal for H3K27ac and H3K27la at promoter regions (TSS ± 5 kb) genome‐wide. (F) Venn diagram showing the overlap among differentially expressed genes from transcriptomic data, differentially abundant proteins from proteomic data, and differential H3K27ac/H3K27la peaks from CUT&Tag data, identifying PROS1 as the only common co‐directional target. (G) IGV tracks showing H3K27la enrichment at the PROS1 promoter. (H) CUT&Tag‐qPCR validation of H3K27la enrichment at the PROS1 promoter using two independent primer pairs (#1, #2), normalized to spike‐in chromatin (*n* = 3). One‐way ANOVA followed by Tukey's multiple‐comparisons test was applied. (I) Western blotting of H3K27la levels in IOMM‐Lee treated with increasing concentrations of 2‐DG or Oxamate for 48 h. (J) Western blotting showing restoration of H3K27la levels in IOMM‐LeeR cells treated with 2‐DG (10 mM) or Oxamate (20 mM) combined with exogenous sodium lactate (10 mM) for 48 h. (K) Dual‐luciferase reporter assay of PROS1 promoter activity in IOMM‐Lee and IOMM‐LeeR cells treated with 2‐DG (10 mM), Oxamate (20 mM), or combined with exogenous sodium lactate (10 mM) for 48 h. Firefly luciferase activity was normalized to Renilla luciferase (*n* = 3). One‐way ANOVA followed by Tukey's multiple‐comparisons test was applied. (L) qPCR analysis of PROS1 mRNA levels in cells treated as in (K) (*n* = 3). One‐way ANOVA followed by Tukey's multiple‐comparisons test was applied. (M) ELISA quantification of secreted PROS1 protein in culture supernatants of cells treated as in (K) (*n* = 3). One‐way ANOVA followed by Tukey's multiple‐comparisons test was applied. (N) Western blot of p16, p21, and p53 in parental cells treated with increasing recombinant PROS1 (0, 30, 60, 90 nM) after 6 Gy irradiation. (O) Representative colony formation under the above conditions (*n* = 3). (P) Quantification of colony formation in (O). One‐way ANOVA followed by Tukey's multiple‐comparisons test was applied. (Q) CCK‐8 cell viability assay under the above conditions (*n* = 3). Two‐way ANOVA test was applied. “n” means the number of individual experiments. Data are presented as mean ± SD. *p < 0.05; **p < 0.01; ***p < 0.001; ns, not significant.

To identify the histone lactylation mark most relevant to radioresistance, we first compared lactylation at multiple histone H3 lysine residues in parental and resistant cells. Resistant cells exhibited markedly increased histone H3 lactylation (Figure 3A, Figure S4B), and site‐specific screening identified H3K27la as the most prominently altered lactylation mark (Figure 3B,C, Figure S4C). Consistent with the dynamic changes observed for global lactylation, H3K27la levels in IOMM‐Lee cells also increased in both a dose‐dependent and time‐dependent manner following irradiation (Figure 3D), indicating that H3K27la is a radiation‐responsive lactylation mark associated with therapy‐induced stress.

We next profiled the genome‐wide distribution of H3K27ac and H3K27la by CUT&Tag sequencing. Heatmap analysis showed substantial enrichment of H3K27la, together with an opposite trend for H3K27ac, in resistant cells (Figure 3E). Integrative analysis of transcriptomic, proteomic, H3K27ac, and H3K27la datasets was performed to identify candidate target genes regulated by both epigenetic modifications and transcriptional output. Briefly, differentially expressed genes (DEGs) from transcriptomic data, differentially expressed proteins (DEPs) from proteomic data, and differential H3K27ac/H3K27la peaks from CUT&Tag sequencing data were intersected using a Venn diagram‐based overlapping strategy. Genes that exhibited co‐directional changes were selected as high‐confidence candidates. Among these, PROS1 was the only gene that satisfied all filtering criteria (Figure 3F). IGV tracks further showed specific enrichment of H3K27la at the PROS1 promoter (Figure 3G), suggesting that PROS1 may be transcriptionally regulated by H3K27la.

We next performed CUT&Tag‐qPCR to quantitatively validate H3K27la occupancy at the PROS1 promoter. Using two independent primer pairs and spike‐in normalization, we found that H3K27la enrichment at the PROS1 promoter was significantly higher in IOMM‐LeeR cells than in parental cells (Figure 3H).

To confirm that glycolysis‐derived lactate regulates H3K27la levels, we treated IOMM‐LeeR cells with 2‐DG or oxamate. Both inhibitors markedly reduced H3K27la expression (Figure 3I). Importantly, co‐incubation with exogenous sodium lactate effectively restored H3K27la levels to those observed before inhibitor treatment (Figure 3J), demonstrating that lactate availability is a limiting factor for H3K27la maintenance.

To determine whether this epigenetic modification functionally drives PROS1 transcription, we conducted a dual‐luciferase reporter assay. Promoter activity was markedly elevated in IOMM‐LeeR cells, suppressed by 2‐DG or Oxamate, and restored by lactate supplementation (Figure 3K). Consistently, qPCR and ELISA analyses demonstrated that PROS1 mRNA and secreted protein levels followed the same pattern (Figure 3L,M). Similar results were obtained in MN2202 cells, confirming the conservation of this regulatory mechanism (Figure S4D,E). These findings indicate that glycolysis‐derived lactate promotes PROS1 expression and secretion through H3K27 lactylation.

To evaluate the functional role of PROS1, we treated irradiated parental cells with recombinant PROS1. Exogenous PROS1 reduced p16, p21, and p53 levels in a dose‐dependent manner (Figure 3N), while enhancing clonogenic survival and proliferative capacity (Figure 3O–Q). These findings were further validated in MN2202 and MN2208 cells (Figure S4F–L), confirming that PROS1‐driven senescence suppression is a conserved feature across multiple meningioma models.

Together, these data indicate that glycolysis‐driven H3K27 lactylation promotes PROS1 expression and secretion in radioresistant cells, and that PROS1 in turn attenuates senescence‐associated phenotypes.

### 2.4 PROS1 Promotes TIS Escape via Receptor TYRO3

**FIGURE 4:**
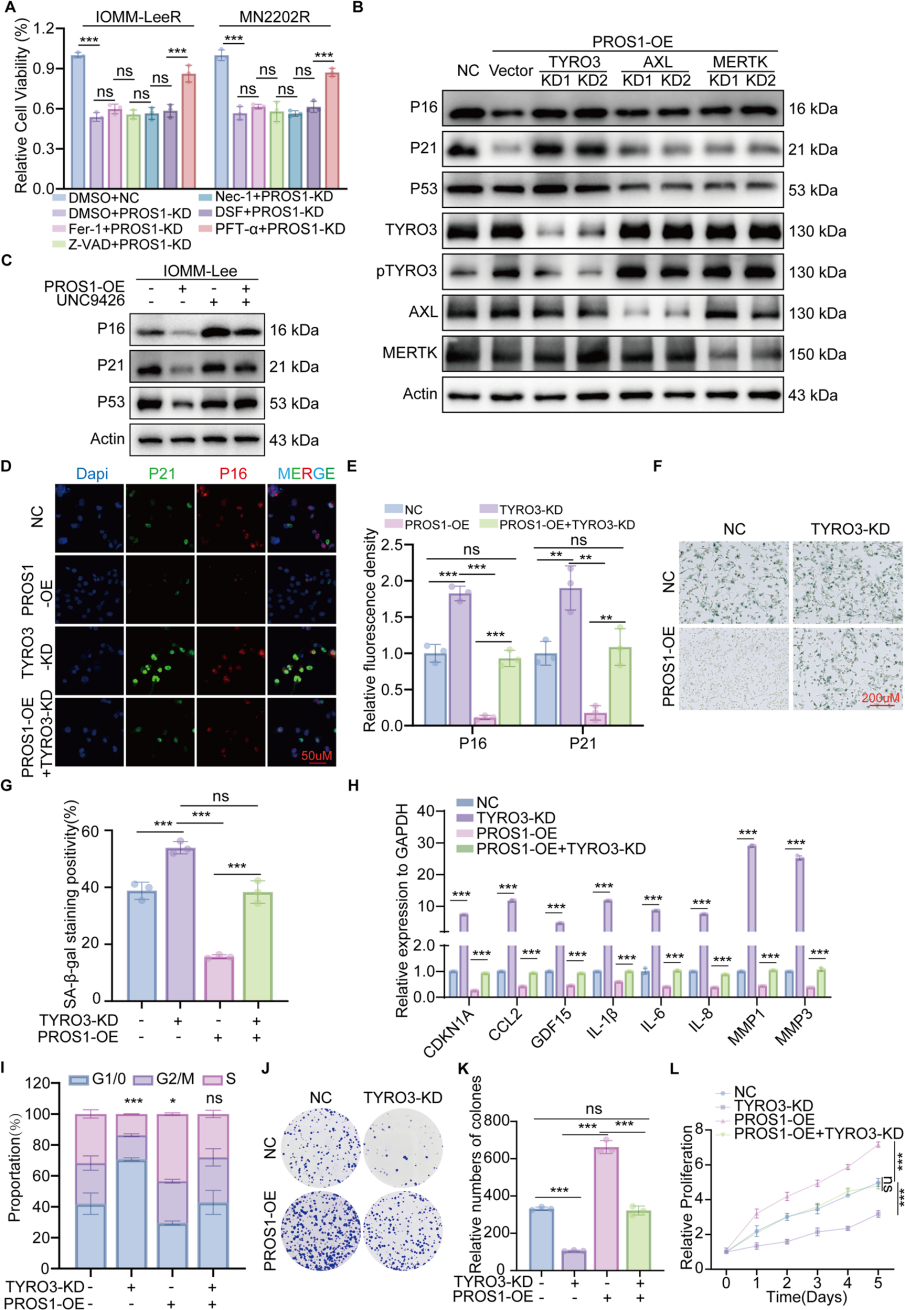
PROS1 Promotes TIS Escape via Receptor TYRO3. (A) CCK‐8 cell viability assay of PROS1‐KD IOMM‐Lee cells treated with various cell death inhibitors for 48 h: Fer‐1 (ferrostatin‐1, 5 µM), Z‐VAD‐FMK (Z‐Val‐Ala‐Asp‐fluoromethylketone, 25 µM), Nec‐1 (necrostatin‐1, 10 µM), DSF (disulfiram, 1 µM), and PFT‐α (pifithrin‐α, 10 µM). Cell viability was normalized to control (DMSO) group (*n* = 3). One‐way ANOVA followed by Tukey's multiple‐comparisons test was applied. (B) Western blot of p16, p21, and p53 in IOMM‐Lee cells with NC, PROS1‐OE, PROS1‐OE + AXL‐KD, PROS1‐OE + TYRO3‐KD, or PROS1‐OE + MERTK‐KD at D15 post‐8 Gy. (C) Western blot of p16, p21, and p53 in IOMM‐Lee cells with NC, PROS1‐OE, UNC9426 (TYRO3 inhibitor, 200 nM). PROS1‐OE + UNC9426 at D15 post‐8 Gy. (D) Immunofluorescence of p21 (green) and p16 (red) in NC, PROS1 OE, TYRO3 KD, and PROS1 OE + TYRO3 KD groups at D15 post‐8 Gy. Scale bar: 50 µm. (E) Quantification of p16 and P21 fluorescence intensity (*n* = 3). One‐way ANOVA followed by Tukey's multiple‐comparisons test was applied. (F) Representative SA‐β‐gal staining of the four groups at D15 post‐8 Gy. Scale bar: 200 µm. (G) Quantification of SA‐β‐gal‐positive cells (*n* = 3). One‐way ANOVA followed by Tukey's multiple‐comparisons test was applied. (H) qPCR of SASP factors in the four groups (*n* = 3). One‐way ANOVA followed by Tukey's multiple‐comparisons test was applied. (I) Flow cytometric cell cycle distribution in the four groups (G0/G1 phase compared, *n* = 3). One‐way ANOVA followed by Tukey's multiple‐comparisons test was applied. (J) Representative colony formation in the four groups. (K) Quantification of colony formation (*n* = 3). One‐way ANOVA followed by Tukey's multiple‐comparisons test was applied. (L) CCK‐8 cell viability assay for the indicated four groups (n = 3). Two‐way ANOVA test was applied. “n” means the number of individual experiments. Data are presented as mean ± SD. *p < 0.05; **p < 0.01; ***p < 0.001; ns, not significant.

Having observed that exogenous recombinant PROS1 promoted cell viability, we next knocked down PROS1 and found that its depletion markedly reduced cell survival, prompting us to perform a pharmacological inhibitor screen to determine whether this growth suppression was attributable to senescence rather than canonical cell death pathways. A panel of agents targeting distinct cellular outcomes was applied, including Fer‐1 (ferroptosis), Z‐VAD‐FMK (apoptosis), Nec‐1 (necroptosis), DSF (cuproptosis), and PFT‐α (a p53 inhibitor that suppresses p53‐mediated senescence). Among all inhibitors tested, only PFT‐α significantly restored the reduced cell viability caused by PROS1 knockdown (Figure 4A). To further support this conclusion at the protein level, we examined representative markers of several regulated cell death pathways. Western blotting showed no consistent activation pattern of ferroptosis‐related markers (GPX4 and ACSL4), apoptosis‐related marker (BAX), necroptosis‐related markers (p‐MLKL and MLKL), or cuproptosis‐related markers (FDX1 and DLAT) in PROS1‐knockdown cells, whereas senescence markers indicated an increased senescent phenotype upon PROS1 knockdown(Figure S5A), further arguing against a predominant contribution of these cell death programs to the observed phenotype.

To identify the receptor responsible for PROS1 signaling, we knocked down the TAM family receptors TYRO3, AXL, and MERTK in PROS1‐overexpressing cells. Among these receptors, only TYRO3 knockdown significantly reversed the PROS1‐induced downregulation of p16, p21, and p53 (Figure 4B; Figure S5B), indicating that PROS1 primarily signals through TYRO3 to mediate TIS escape. To further confirm that PROS1‐induced TIS escape requires TYRO3 kinase activity, we treated PROS1‐OE cells with the selective TYRO3 inhibitor UNC9426. Co‐treatment with UNC9426 effectively restored p16, p21, and p53 expression (Figure 4C, Figure S5C).

We then generated cell lines with PROS1 overexpression, TYRO3 knockdown, or the corresponding combined modifications. Immunofluorescence showed that PROS1 overexpression reduced p16 and p21 expression, whereas TYRO3 knockdown increased their levels; importantly, TYRO3 depletion restored p16 and p21 expression in PROS1‐overexpressing cells. These effects were consistently supported by SA‐β‐gal staining, qPCR analysis of SASP factors, flow cytometric cell cycle analysis, clonogenic assays, and CCK‐8 assays (Figure 4D–L; Figure S6A–G). Collectively, these findings indicate that PROS1 promotes TIS escape predominantly through TYRO3 activation.

### 2.5 TYRO3 Interacts With CARF

**FIGURE 5:**
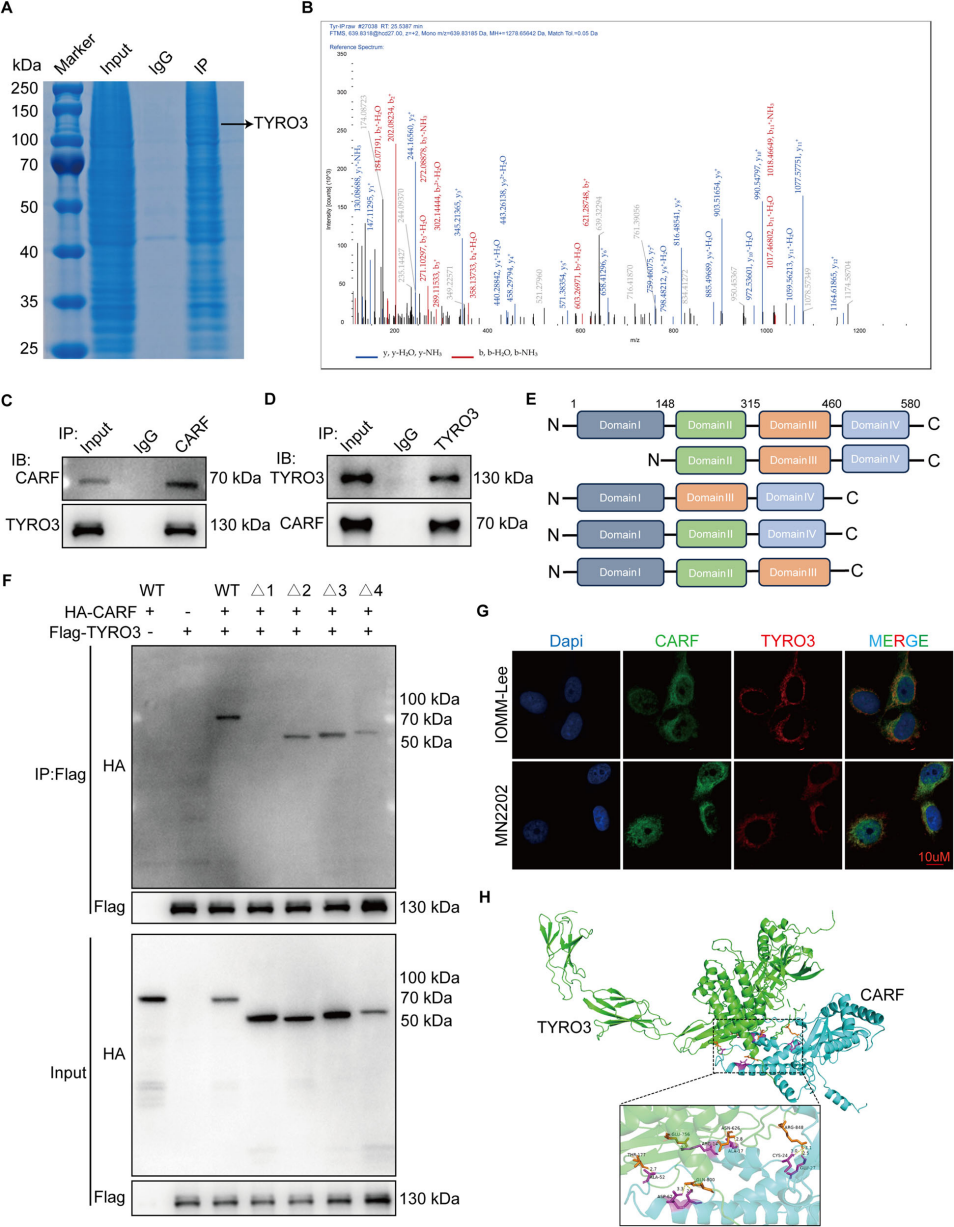
TYRO3 Interacts with CARF. (A) SDS‐PAGE Coomassie blue staining of IOMM‐Lee lysates immunoprecipitated with anti‐TYRO3 or control IgG, showing specific bands. (B) Mass spectrometry identification of CARF (CDKN2AIP) in TYRO3 immunoprecipitates; representative MS/MS spectrum. (C, D) Co‐IP validation of endogenous TYRO3‐CARF interaction using anti‐HA‐CARF (C) or anti‐Flag‐TYRO3 (D) antibodies. (E) Schematic of CARF truncation constructs: full‐length (1‐580 aa) and four truncation mutants (1‐148, 149–315, 316–460, 461–580), all HA‐tagged. (F) Co‐IP of each CARF truncation with TYRO3, mapping the 1–148 aa region as essential for interaction. (G) Confocal microscopy showing co‐localization of endogenous CARF (green) and TYRO3 (red) in IOMM‐Lee (top) and MN2202 (bottom) cells. DAPI (blue). Scale bar: 10 µm. (H) Molecular docking model of TYRO3‐CARF binding. Top: overall complex; bottom: enlarged interface showing key interacting residues. Visualized with PyMOL.

To identify downstream effectors through which TYRO3 mediates TIS escape, we performed immunoprecipitation‐mass spectrometry (IP‐MS). In IOMM‐Lee cells, Coomassie blue staining and mass spectrometric analysis of TYRO3 immunoprecipitates identified CARF (CDKN2AIP) as a candidate interacting protein (Figure 5A,B). Co‐immunoprecipitation further confirmed an endogenous interaction between TYRO3 and CARF in multiple meningioma cell lines (Figure 5C,D), supporting CARF as a downstream binding partner of TYRO3. Using CARF truncation constructs, we mapped the N‐terminal region (amino acids 1–148) as the domain required for TYRO3 binding (Figure 5E,F). Confocal microscopy showed co‐localization of TYRO3 and CARF, and molecular docking further supported the structural basis of this interaction (Figure 5G,H; Figure S7A,B). Together, these data identify CARF as a TYRO3‐interacting protein and suggest that it may serve as a downstream effector linking TYRO3 signaling to TIS escape.

### 2.6 TYRO3 Promotes CARF Tyrosine 8 Phosphorylation and Degradation to Facilitate TIS Escape

**FIGURE 6:**
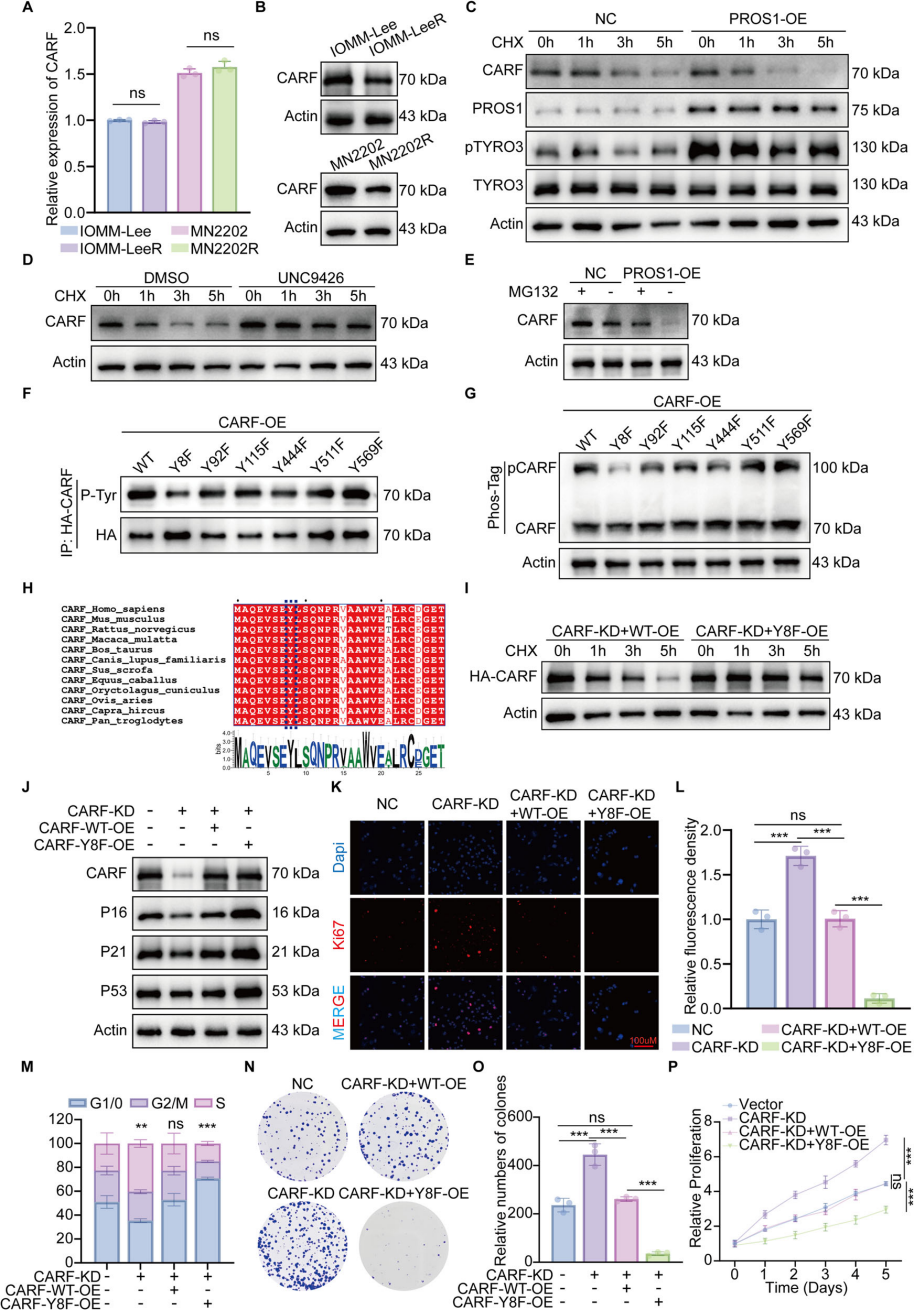
TYRO3 Promotes CARF Tyrosine 8 Phosphorylation and Degradation to Facilitate TIS Escape. (A) qPCR of CARF mRNA in parental (IOMM‐Lee, MN2202) and radioresistant (IOMM‐LeeR, MN2202R) cells (*n* = 3). Unpaired two‐tailed Student's t‐test was applied. (B) Western blotting of CARF protein levels in parental and radioresistant cells of both lines. (C) Cycloheximide (CHX) chase assay of CARF protein stability in control and PROS1‐OE IOMM‐Lee cells. Cells were treated with CHX (100 µg/mL) for 0, 1, 3, and 5 h, and CARF protein levels were detected by western blotting. (D) CHX chase assay of CARF protein stability in IOMM‐Lee cells treated with DMSO or UNC9426 (200 nM). (E) CHX + MG132 treatment for 5 h, Western blot confirming proteasomal degradation of CARF. (F) Mutation of six individual tyrosine residues in CARF to phenylalanine (Y8F, Y42F, Y103F, Y157F, Y211F, Y289F), immunoprecipitation with anti‐HA followed by anti‐phosphotyrosine (p‐Tyr) detection. (G) Phos‐tag SDS‐PAGE of CARF wild‐type (WT) and mutants showing phosphorylation mobility shifts. (H) Multi‐species sequence alignment of CARF N‐terminus showing highly conserved Y8. (I) CHX chase in CARF‐KD cells reconstituted with WT or Y8F CARF, anti‐HA detection. (J) Western blot of p16, p21, and p53 in the four groups. (K) Immunofluorescence of Ki67 (red) with DAPI (blue) in the four groups. Scale bar: 100 µm. (L) Quantification of Ki67 fluorescence intensity (*n* = 3). One‐way ANOVA followed by Tukey's multiple‐comparisons test was applied. (M) Flow cytometry cell cycle distribution at D15 post‐8 Gy for NC, CARF‐KD, CARF‐KD + WT‐OE, and CARF‐KD + Y8F‐OE groups (G0/G1 phase compared; *n* = 3). One‐way ANOVA followed by Tukey's multiple‐comparisons test was applied. (N, O) Representative colony formation and quantification in the four groups. (*n* = 3). One‐way ANOVA followed by Tukey's multiple‐comparisons test was applied. (P) CCK‐8 cell viability assay for the indicated groups (*n* = 3). Two‐way ANOVA test was applied. “n” means the number of individual experiments. Data are presented as mean ± SD. *p < 0.05; **p < 0.01; ***p < 0.001; ns, not significant.

Although CARF mRNA levels were comparable between parental and resistant cells in both IOMM‐Lee and MN2202 pairs (Figure 6A), CARF protein levels were markedly lower in both radioresistant sublines (Figure 6B), consistent with our transcriptomic and proteomic datasets.

We next investigated whether PROS1‐TYRO3 signaling regulates CARF protein stability. Cycloheximide (CHX) chase assays showed that PROS1 overexpression markedly shortened the half‑life of CARF protein compared to control cells (Figure 6C; Figure S7E). Conversely, treatment with the selective TYRO3 inhibitor UNC9426 significantly extended the half‑life of endogenous CARF protein compared to DMSO control (Figure 6D), confirming that basal TYRO3 kinase activity contributes to constitutive CARF turnover. Furthermore, the proteasome inhibitor MG132 blocked PROS1‑OE‑induced CARF degradation (Figure 6E), and ubiquitination assays confirmed that PROS1‑OE markedly increased the ubiquitination level of CARF in both IOMM‑Lee and MN2202 cells (Figure S7F), implicating the ubiquitin‐proteasome pathway in this process.

To understand how CARF exerts its senescence‐regulatory function, we examined its interaction with key senescence‐associated proteins. Co‐immunoprecipitation (Co‐IP) experiments revealed that endogenous CARF physically interacts with both p16 and p53 in IOMM‐Lee cells (Figure S7C), suggesting that CARF may coordinate senescence signaling through protein‐protein interactions. CHX chase assays further showed that in CARF‐knockdown cells, the protein half‐lives of both p16 and p53 were significantly shortened compared to control cells (Figure S7D), indicating that CARF is required for maintaining the stability of these key senescence regulators.

Because TYRO3 is a receptor tyrosine kinase, we next examined whether CARF phosphorylation is associated with TYRO3 activity. Individual mutation of the six tyrosine residues in CARF revealed that the Y8F mutant (Tyr8 to Phe) exhibited an almost complete loss of tyrosine phosphorylation (Figure 6F). Phos‐tag SDS‐PAGE further confirmed markedly reduced phosphorylation of the Y8F mutant (Figure 6G). Sequence alignment showed that Tyr8 is highly conserved across species (Figure 6H), and protein stability assays demonstrated that the Y8F mutant had a significantly longer half‐life than wild‐type CARF (Figure 6I; Figure S7I), indicating that phosphorylation at this site is critical for CARF degradation.

To determine the functional consequences of CARF destabilization, we re‐expressed wild‐type CARF or the degradation‐resistant Y8F mutant in CARF‐knockdown cells. Wild‐type CARF partially restored p16, p21, and p53 expression as well as G0/G1 arrest, whereas the Y8F mutant exerted stronger tumor‐suppressive effects, as evidenced by increased senescence marker expression, reduced Ki67 positivity, and markedly impaired clonogenic and proliferative capacity (Figure 6J–P; Figures S7G,H,J and S8A–G). Overall, these results indicate that TYRO3‐dependent phosphorylation of CARF at tyrosine 8 promotes its proteasomal degradation, thereby relieving p14ARF/p53‐associated senescence and facilitating TIS escape.

### 2.7 Targeting the PROS1/CARF Axis Restores Radiosensitivity in Vivo

**FIGURE 7:**
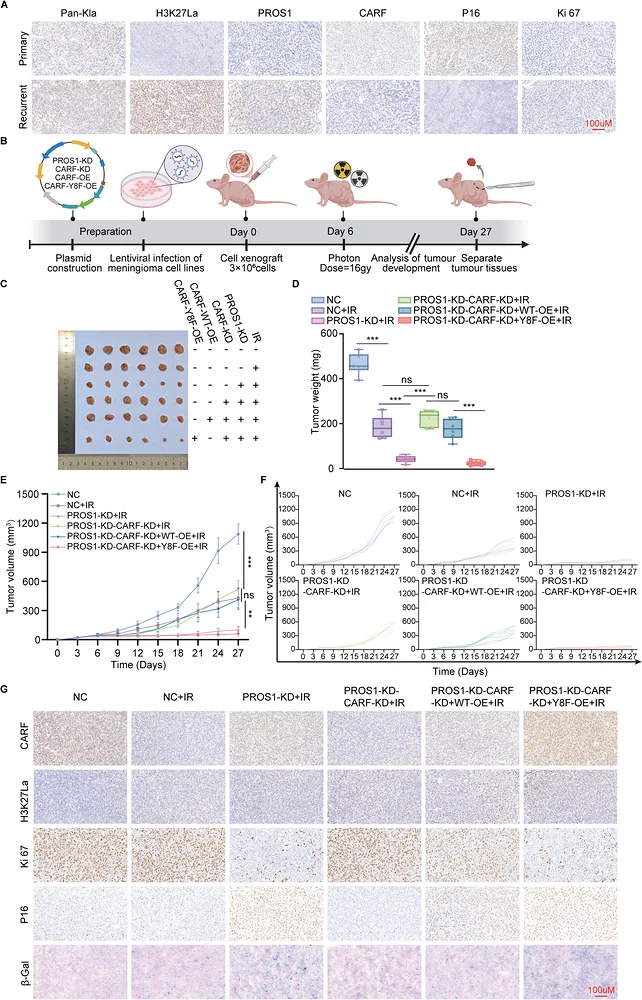
Targeting the PROS1/CARF Axis Restores Radiosensitivity In Vivo. (A) Representative IHC images of Pan‐Kla, H3K27la, PROS1, CARF, p16, and Ki67 in radiotherapy‐naive primary meningiomas versus post‐irradiation recurrent meningiomas (N = 6/group). Scale bar: 100 µm. (B) Experimental scheme for subcutaneous xenograft: IOMM‐LeeR stable lines with indicated modifications were implanted in BALB/c nude mice (N = 6/group); a single local dose of irradiation (16 Gy) was given on day 6; tumors harvested on day 27. Created with BioRender.com, agreement number: MT2A1FMWGM. (C) Representative tumor images. Groups: NC, NC + IR, PROS1‐KD + IR, PROS1‐KD + CARF‐KD + IR, PROS1‐KD + CARF‐KD + WT‐OE + IR, and PROS1‐KD + CARF‐KD + Y8F‐OE + IR. (D) Ex vivo tumor weights per group (N = 6/group). One‐way ANOVA followed by Tukey's multiple‐comparisons test was applied. (E) Tumor growth curves (day 0–27) (N = 6/group). Two‐way ANOVA test was applied. (F) Individual tumor growth curves for each mouse in the indicated groups. (G) Representative images of IHC for CARF, H3K27La, Ki67, P16, and SA‐β‐gal staining in tumor tissues from the indicated groups. Scale bar: 100 µm. “N” means the number of subjects. Data are presented as mean ± SD. *p < 0.05; **p < 0.01; ***p < 0.001; ns, not significant.

To extend the translational relevance of our findings to clinical settings, we collected a cohort of radiotherapy‐naive primary meningiomas and post‐irradiation recurrent meningiomas that had progressed after photon‐based radiotherapy. IHC analysis of these specimens revealed that recurrent tumors exhibited markedly elevated Pan‐Kla, H3K27la, and PROS1 expression compared with primary tumors, accompanied by a reciprocal reduction in CARF protein levels. In parallel, p16 staining was significantly decreased in the recurrent group, consistent with the loss of senescence‐associated markers, whereas Ki67 positivity was substantially increased, reflecting enhanced proliferative capacity (Figure 7A; Figure S9A).

We next examined the in vivo relevance of this pathway using a subcutaneous xenograft model in nude mice (Figure 7B). In IOMM‐LeeR‐bearing mice, radiotherapy significantly inhibited tumor growth. PROS1 knockdown further enhanced this response, as reflected by marked reductions in tumor volume and weight, whereas additional CARF knockdown abolished the radiosensitizing effect of PROS1 depletion. Notably, in the context of PROS1 knockdown, overexpression of the degradation‐resistant CARF Y8F mutant rendered tumors highly radiosensitive and significantly reduced tumor burden (Figure 7C–F).

Histological and staining analyses further supported these in vivo effects. SA‐β‐gal staining and immunohistochemical analysis demonstrated that PROS1 knockdown effectively restored senescence‑associated phenotypes in irradiated tumors. As shown in Figure 7G, xenografts from the PROS1‑KD + IR group exhibited increased SA‐β‑gal positivity and elevated p16 and p53 expression, accompanied by reduced Ki67 staining compared with the NC + IR group, indicating that PROS1 depletion re‑instates therapy‑induced senescence in vivo. Notably, concurrent CARF knockdown largely attenuated these effects, whereas re‑expression of the degradation‑resistant CARF Y8F mutant, but not wild‑type CARF, significantly restored senescence‑associated phenotypes and suppressed proliferative activity (Figure 7G). In addition, Pan‑Kla, H3K27la, and PROS1 levels were markedly elevated in irradiated groups, while CARF protein displayed a reciprocal reduction. Notably, pTYRO3 signals closely paralleled PROS1 abundance across all groups, supporting a functional coupling between PROS1 secretion and TYRO3 activation (Figure S9B,C). Collectively, these in vivo findings demonstrate that disrupting PROS1 signaling or stabilizing CARF effectively restores senescence and overcomes radioresistance in meningioma, reinforcing the therapeutic potential of targeting this axis.

### 2.8 Proposed Model of the PROS1‐TYRO3‐CARF Axis Driving TIS Escape and Radioresistance

**FIGURE 8:**
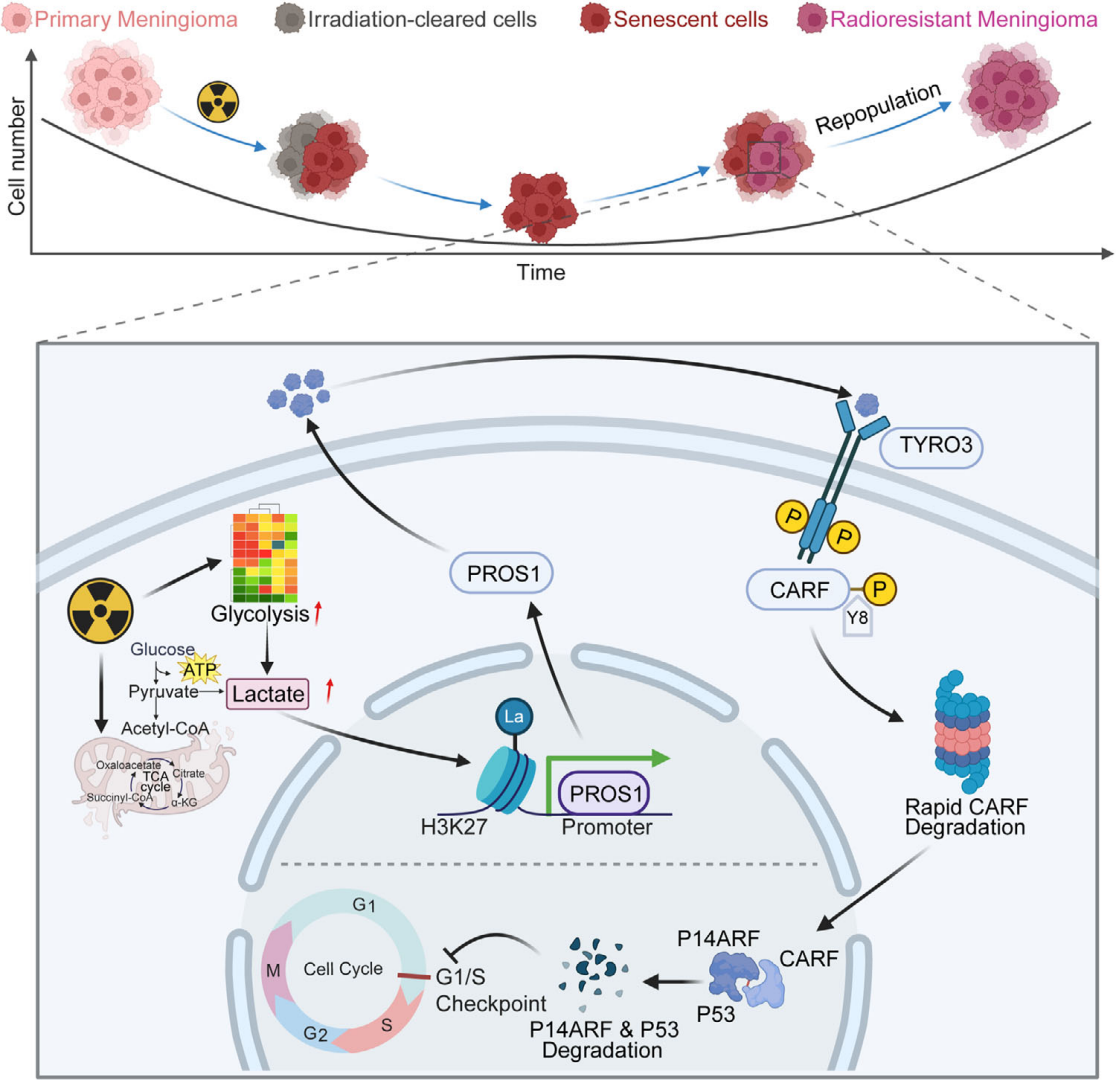
Proposed Model of the PROS1‐TYRO3‐CARF Axis Driving TIS Escape and Radioresistance. Following radiotherapy, a substantial fraction of tumor cells is eliminated, whereas a surviving subset undergoes therapy‐induced senescence and subsequently re‐enters the cell cycle through TIS escape, thereby repopulating as radioresistant cells. In radioresistant meningioma cells, enhanced glycolysis promotes lactate accumulation and increased H3K27 lactylation. H3K27la is enriched at the PROS1 promoter, transcriptionally activating PROS1 expression and secretion. Secreted PROS1 activates TYRO3 in a paracrine manner, and activated TYRO3 binds CARF and promotes its phosphorylation at tyrosine 8, triggering proteasomal degradation of CARF. Loss of CARF relieves p14ARF/p53‐mediated senescence, thereby enabling TIS escape and the subsequent repopulation of radioresistant tumor cells. Key nodes in this pathway, including PROS1, TYRO3, and CARF Y8 phosphorylation, represent potential therapeutic targets for overcoming radioresistance. Created with BioRender.com, agreement number: QI2A1FN4GQ.

Based on these findings, we propose a model in which glycolysis‐driven H3K27 lactylation promotes PROS1 expression and secretion, leading to TYRO3 activation, CARF degradation, TIS escape, and ultimately radioresistance in meningioma (Figure 8).

## 3 Discussion

In the present study, we identified a novel glycolysis‐H3K27 lactylation‐PROS1‐TYRO3‐CARF signaling axis that drives radioresistance in meningioma. Our data support a model in which radioresistant meningioma cells undergo metabolic reprogramming toward enhanced glycolysis, resulting in lactate accumulation and increased H3K27 lactylation. This epigenetic change promotes transcriptional upregulation of PROS1, whose secretion activates TYRO3 and triggers phosphorylation‐dependent degradation of CARF, thereby relieving p14ARF/p53‐associated senescence and facilitating escape from TIS. By linking metabolic adaptation, chromatin remodeling, receptor tyrosine kinase signaling, and TIS escape within a unified mechanistic framework, this study provides new insight into how meningioma cells survive and adapt under radiotherapeutic stress.

One of the central findings of this work is that glycolysis‐derived lactate can influence radioresistance by regulating the transcription of a secreted signaling factor through histone lactylation. Although previous studies have emphasized the role of lactylation in immune regulation and intracellular metabolic adaptation, our results extend the functional relevance of this modification to the control of tumor‐derived secretory programs [35]. In a similar vein, our conditioned medium experiments indicated that SASP factors, another class of secreted molecules, also promoted radioresistance in a paracrine manner. These observations underscore the potential importance of secreted proteins in shaping the resistant phenotype and suggest that PROS1 may be part of a broader secretory program that supports TIS escape. In particular, the identification of PROS1 as an H3K27la‐associated transcriptional target suggests that lactylation‐dependent chromatin remodeling may influence not only cell‐intrinsic stress responses but also extracellular communication pathways that reinforce treatment resistance. These findings expand the current understanding of histone lactylation from a metabolic epigenetic mark to a regulator of tumor‐promoting ligand‐receptor signaling [36]. Notably, H3K27la enrichment at the PROS1 promoter appears to be highly selective, implying that local chromatin context, associated cofactors, or specific reader proteins may confer locus‐specific responsiveness to lactate‐driven epigenetic regulation [37, 38, 39]. In addition, our untargeted metabolomic analysis revealed broader metabolic rewiring in radioresistant meningioma cells, including alterations in central carbon and nucleotide metabolism. These findings suggest that TIS escape is accompanied by a coordinated metabolic adaptation rather than a glycolytic change alone, although our study focused on the lactate‐H3K27la‐PROS1 axis.

Another important aspect of our study is the identification of TYRO3 as the predominant downstream receptor mediating PROS1‐dependent TIS escape in radioresistant meningioma cells. Previous studies of the PROS1/TAM pathway in cancer have focused primarily on AXL and MERTK, especially in relation to tumor progression, metastasis, and resistance to systemic therapy, whereas TYRO3 has received comparatively less attention [40]. Our findings indicate that in the context of radiotherapy‐induced senescence, PROS1 preferentially signals through TYRO3 to promote escape from growth arrest. This observation is consistent with recent structural evidence showing specific contacts between the LG1 domain of PROS1 and the first immunoglobulin domain of TYRO3, stabilized by electrostatic, hydrophobic, and polar interactions [41]. Such structural specificity may provide a molecular basis for the preferential engagement of TYRO3 in this context [42]. At the same time, TAM receptors are known to function in a coordinated rather than strictly independent manner, and the potential formation of TYRO3‐MERTK heterodimers raises the possibility that receptor crosstalk further shapes signaling output in meningioma [43]. In addition, our IP‐MS analysis identified a distinct TYRO3‐associated protein network, including CARF, SIRT6, KIF20A, and MCM family members, supporting the idea that TYRO3 may possess non‐redundant functions in cell cycle regulation and DNA damage responses [44, 45]. Consistently, pharmacological inhibition of TYRO3 using the selective inhibitor UNC9426 effectively restored CARF protein levels and reversed PROS1‐induced suppression of senescence markers, further confirming that TYRO3 kinase activity is essential for PROS1‐driven TIS escape [46]. Given that selective inhibitors of TYRO3 remain underdeveloped compared with those targeting AXL or MERTK, our findings provide a rationale for further exploration of TYRO3‐directed therapeutic strategies in radioresistant tumors.

Mechanistically, our study also reveals CARF as a downstream substrate of TYRO3 and identifies tyrosine 8 as a critical phosphorylation site governing CARF stability. CARF has previously been implicated in p14ARF‐dependent tumor suppression and senescence regulation, but its upstream regulatory mechanisms have remained poorly defined [47]. Here, we demonstrate that CARF interacts with p16 and p53 and is required for maintaining their protein stability, as CARF knockdown significantly shortened their half‑lives. Moreover, ubiquitination assays confirmed that PROS1 overexpression promotes CARF polyubiquitination, indicating that CARF is subjected to ubiquitin‐proteasome degradation downstream of PROS1‐TYRO3 signaling. We show that TYRO3 interacts with CARF, promotes its phosphorylation at tyrosine 8, and triggers its proteasomal degradation, thereby connecting extracellular receptor signaling to the intracellular machinery that maintains senescence. This finding provides a mechanistic explanation for how radioresistant cells may overcome senescence‐associated growth arrest despite persistent therapeutic stress. Importantly, the functional relevance of this site was supported by truncation mapping, point mutation analysis, phosphorylation assays, and rescue experiments. The strong evolutionary conservation of tyrosine 8 further underscores its biological importance. Because this residue lies within the N‐terminal disordered region of CARF, it is plausible that phosphorylation at this position facilitates recognition by ubiquitin ligases or other components of the proteasomal degradation machinery [48]. Although the precise degradation complex remains to be identified, our data establish phosphorylation‐dependent destabilization of CARF as a key event in TIS escape.

Beyond its mechanistic significance, this signaling axis may also have translational implications. Our in vivo experiments show that interfering with PROS1 or preventing CARF destabilization restores senescence‐associated phenotypes and enhances tumor radiosensitivity, suggesting that this pathway may be therapeutically actionable. From a clinical perspective, several components of this axis may have potential value as biomarkers or intervention points. Elevated glycolytic activity, increased H3K27la, high PROS1 expression, enhanced TYRO3 activation, or reduced CARF stability may each reflect a biological state associated with resistance to radiotherapy. More broadly, these observations support the concept that metabolic adaptation and TIS escape are not merely parallel features of resistant tumors but are mechanistically integrated processes. Targeting this integration may therefore represent a promising strategy for radiosensitization in meningioma.

Several limitations of this study should be acknowledged. First, while we validated the core findings in two independently established radioresistant models (IOMM‐LeeR and MN2202R) and in additional patient‐derived cell lines (MN2202 and MN2208), the activity and clinical relevance of this pathway may still differ across molecular subtypes, tumor grades, and primary patient samples, and thus require validation in broader clinical cohorts. Moreover, the subcutaneous xenograft model used in this study, while valuable for mechanistic validation, does not fully recapitulate the intracranial or meningeal microenvironment, the anatomical context of radiotherapy, or the immune components that may contribute to meningioma radioresistance. Second, because meningiomas often contain abundant tumor‐associated macrophages and other immune cell populations, it will be important to determine whether the PROS1/TYRO3 axis also influences senescence‐related phenotypes within the tumor microenvironment, particularly in macrophages or T cells [49]. In addition, although our Co‐IP and colocalization data support a strong association between TYRO3 and CARF, definitive proof of a direct physical interaction would require purified protein pull‐down assays or surface plasmon resonance analyses. Third, the epigenetic relationship between H3K27la and H3K27ac warrants further clarification. These two marks may share common writers and erasers, suggesting potential competition and dynamic switching at functionally relevant loci. p300/CBP have been implicated as shared writers, whereas HDAC and SIRT family proteins may act as shared erasers, raising the possibility that cellular senescence and lactate accumulation favor a shift from H3K27ac to H3K27la [50]. While our study focused on histone H3K27la, we cannot exclude the possibility that non‐histone protein lactylation may also contribute to the observed phenotypes; systematic lactylome profiling would be an important direction for future investigations [51]. Defining whether such a modification switch occurs at single‐locus or genome‐wide levels, and how it contributes to TIS escape, will require more refined approaches such as sequential chromatin profiling or single‐cell epigenomic analyses.

In conclusion, this study identifies the glycolysis‐H3K27 lactylation‐PROS1‐TYRO3‐CARF axis as a novel metabolic‐epigenetic‐signaling mechanism underlying meningioma radioresistance. Mechanistically, enhanced glycolysis and lactate accumulation promote H3K27 lactylation‐dependent PROS1 transcription, while secreted PROS1 activates TYRO3 to drive phosphorylation‐dependent degradation of CARF, thereby relieving senescence‐associated cell cycle arrest, facilitating TIS escape, and ultimately promoting resistance to radiotherapy. By demonstrating how metabolic reprogramming is coupled to TIS escape through epigenetic activation of extracellular signaling and CARF destabilization, this work broadens the current understanding of resistance biology in meningioma and provides mechanistic insight into the adaptive response of tumor cells to radiotherapy. These findings also highlight the PROS1‐TYRO3‐CARF pathway as a promising source of potential biomarkers and therapeutic targets for radiosensitization.

## 4 Experimental Section

### 4.1 Cell Lines and Culture

Human meningioma IOMM‐Lee cells were obtained from the American Type Culture Collection (ATCC CRL‐3370), and the derived radioresistant subline IOMM‐LeeR was established in our laboratory by repeated exposure to fractionated photon irradiation, as described below. In addition, two patient‐derived benign meningioma cell lines, MN2202 and MN2208, were established from freshly resected surgical specimens under Ethics Approval No. KY‐2021‐787. Primary tumor cells were isolated by a combination of mechanical dissociation and enzymatic digestion, followed by short‐term primary culture. To enrich for meningioma cells and remove contaminating non‐tumor cells, the primary cultures were further purified by fluorescence‐activated cell sorting using a positive‐selection strategy. The enriched cells were subsequently immortalized by hTERT transduction, and stable immortalized cell lines were expanded for validation experiments. HEK293T cells (ATCC, CRL‐3216) were used for lentiviral packaging. IOMM‐Lee, IOMM‐LeeR, MN2202, MN2208 and HEK293T cells were cultured in DMEM (Cat. No. 11965092, Gibco, Grand Island, NY, USA) supplemented with 10% fetal bovine serum (Cat. No. A5256701, Gibco, Grand Island, NY, USA) and 1% penicillin‐streptomycin (Cat. No. 15140122, Gibco, Grand Island, NY, USA) at 37°C in a humidified incubator containing 5% CO_2_. Culture medium was replaced every 2–3 days, and cells were passaged at approximately 70%–80% confluence using 0.25% trypsin‐EDTA (Cat. No. 25200072, Gibco, Grand Island, NY, USA). All cells used for experiments were maintained in the logarithmic growth phase. The identity of MN2202 and MN2208 cells was confirmed by immunofluorescence staining for meningioma‐associated markers, including vimentin and epithelial membrane antigen (EMA), as shown in Figure S1A. All cell lines were routinely tested and confirmed to be free of mycoplasma contamination before use.

### 4.2 Establishment of Radioresistant Cell Line

Irradiation was delivered using a 6 MV x‐ray linear accelerator (Varian TrueBeam) at a dose rate of 2 Gy/min and a source‐to‐surface distance of 100 cm. To establish radioresistant meningioma sublines, parental IOMM‐Lee and MN2202 cells were exposed to a fractionated irradiation regimen consisting of sequential doses of 2, 4, 6, and 8 Gy, with each dose administered five times and a 72‐h recovery interval between irradiations. After completion of each dose level, cells were passaged and allowed to recover for 1 week before the next dose escalation. The total cumulative dose was 100 Gy. Surviving cells were expanded and designated as IOMM‐LeeR and MN2202R, respectively. Parental cells were passaged in parallel and served as controls.

### 4.3 Multi‐Omics Analysis

Transcriptomic analysis: Total RNA was extracted using the RNeasy Kit (Cat. No. 74181, QIAGEN, Hilden, Germany) and sequenced on an Illumina NovaSeq 6000 platform (PE150). Sequencing data were processed using Trimmomatic, aligned with STAR, and analyzed for differential expression with DESeq2 (|log2FC| > 1, p < 0.05). Proteomic analysis: Cell lysates were digested with trypsin, labeled with TMT, and analyzed by LC‐MS/MS on an Orbitrap Fusion Lumos instrument; the resulting data were processed using MaxQuant. Metabolomic analysis: Cell pellets were extracted with 80% methanol and analyzed by LC‐MS/MS on a Q Exactive HF instrument; the resulting data were processed using Compound Discoverer.

### 4.4 Plasmids, Transfections, and Lentivirus Construction

Human full‐length coding sequences of PROS1, TYRO3, and CARF were amplified by PCR from IOMM‐Lee cell cDNA. All overexpression plasmids based on pcDNA3.1(+) or pCDH‐CMV‐MCS‐EF1‐Puro lentiviral vectors, as well as truncation and point mutants, were constructed in‐house. Truncation mutants (CARF 1–148, 149–315, 316–460, and 461–580) and point mutants (Y‐to‐F substitutions: Y8F, Y42F, Y103F, Y157F, Y211F, and Y289F) were generated by homologous recombination using ClonExpress II One Step Cloning Kit (Cat. No. C112, Vazyme, Nanjing, China). shRNA target sequences were synthesized by GeneChem and cloned into the pLKO.1 vector. Transfections were performed using Lipofectamine 3000(Cat. No. L3000015, Invitrogen, Carlsbad, CA, USA). Lentiviral packaging was performed in HEK293T cells (ATCC, CRL‐3216) using psPAX2 and pMD2.G helper plasmids. Stable cell lines were selected with puromycin (Cat. No. P8833, Sigma–Aldrich, St. Louis, MO, USA).

### 4.5 Colony Formation Assays

Cells were seeded at 500–2000 cells per well in 6‐well plates, allowed to adhere overnight, and irradiated with single doses of 0, 2, 4, 6, or 8 Gy. After 10–14 days, with the medium changed every 3 days, colonies were fixed with methanol for 15 min and stained with 0.5% crystal violet for 20 min (Cat. No. 60506ES60, Yeasen Biotechnology, Shanghai, China). Colonies containing at least 50 cells were counted.

### 4.6 CCK‐8 Cell Viability Assay

Cells were seeded at 5 × 10^3^ cells per well in 96‐well plates containing 100 µL of medium, allowed to adhere overnight, and treated as indicated in the experimental design. Cell viability was measured using a CCK‐8 reagent (Cat. No. U22‐001A, YOBIBIO Biotechnology, Shanghai, China). At each indicated time point, 10 µL of CCK‐8 reagent was added to each well, followed by incubation at 37°C in 5% CO_2_ for 1–4 h. Absorbance was measured at 450 nm using a microplate reader.

### 4.7 Cell Death Inhibitor Screening

PROS1‐knockdown cells were seeded in 96‐well plates and treated with the following inhibitors: FER‐1 (Ferrostatin‐1, 5 µM) (Cat. No. HY‐100579, MedChemExpress, Monmouth Junction, NJ, USA), Z‐VAD‐FMK (Z‐Val‐Ala‐Asp‐fluoromethylketone, 25 µM) (Cat. No. HY‐16658, MedChemExpress, Monmouth Junction, NJ, USA), Nec‐1 (Necrostatin‐1, 10 µM) (Cat. No. HY‐15760, MedChemExpress, Monmouth Junction, NJ, USA), DSF (Disulfiram, 1 µM) (Cat. No. HY‐B0240, MedChemExpress, Monmouth Junction, NJ, USA), and PFT‐α (pifithrin‐α, 10 µM) (Cat. No. HY‐15484, MedChemExpress, Monmouth Junction, NJ, USA). Cell viability was assessed by CCK‐8 assay after 48 h.

### 4.8 Transmission Electron Microscopy (TEM)

Approximately 1 × 10^6^ cells were fixed with EM‐grade glutaraldehyde (Cat. No. G5882, Sigma–Aldrich, St. Louis, MO, USA) for 2 h at 4°C, washed three times with PBS, and post‐fixed with 1% osmium tetroxide (Cat. No. 18451, Ted Pella, Redding, CA, USA) for 1 h at 4°C. After graded ethanol dehydration (30%, 50%, 70%, 90%, and 100%), the samples were embedded in Embed‐812 epoxy resin (Cat. No. 14120, Electron Microscopy Sciences, Hatfield, PA, USA). Ultrathin sections (70 nm; Leica UC7) were stained with uranyl acetate and lead citrate and imaged using a transmission electron microscope (Hitachi HT7800, Japan) at 80 kV. Images were acquired using an AMT camera.

### 4.9 Scanning Electron Microscopy (SEM)

Cells grown on coverslips were fixed with 2.5% glutaraldehyde (Cat. No. G5882, Sigma–Aldrich, St. Louis, MO, USA), dehydrated through a graded ethanol series, critical‐point dried (Leica EM CPD300), and sputter‐coated with gold (Hitachi E‐1010 Ion Sputter). Samples were examined using a scanning electron microscope (Hitachi SU8010, Japan) at 5–10 kV, and five random fields were acquired for each sample.

### 4.10 SA‐β‐Gal Staining

SA‐β‐gal staining was performed using a senescence β‐galactosidase staining kit (Cat. No. C0602, Beyotime, Shanghai, China) according to the manufacturer's instructions. For cultured cells, cells in 6‐ or 24‐well plates were fixed at the indicated time points, washed, and incubated overnight at 37°C without CO_2_ in staining solution containing X‐gal (pH 6.0). At least 200 cells per well were counted across five random fields, and blue‐positive cells were scored as senescent. For tumor tissues, freshly excised xenograft tumors were embedded in OCT compound, cryosectioned, and subjected to SA‐β‐gal staining according to the same kit protocol. Stained frozen sections were imaged and analyzed under a light microscope.

### 4.11 Western Blotting and Antibodies

For detection of total cellular proteins, cells were lysed in RIPA buffer (Cat. No. 89900, Thermo Fisher Scientific, Waltham, MA, USA) supplemented with a protease inhibitor cocktail (Cat. No. 4693116001, Roche, Basel, Switzerland) and a phosphatase inhibitor (Cat. No. 4906837001, Roche, Basel, Switzerland). For detection of histone lactylation and pan‐lactylation signals, cells were lysed directly in SDS lysis buffer supplemented with protease and phosphatase inhibitors. This lysis approach enables efficient enrichment of protein signals specifically within the histone‐enriched nuclear fraction [52]. Protein concentrations were determined using a BCA assay kit (Cat. No. 23225, Thermo Fisher Scientific, Waltham, MA, USA). Equal amounts of protein were separated by SDS‐PAGE, transferred onto PVDF membranes (Cat. No. IPVH00010, Millipore, Billerica, MA, USA), blocked with 5% nonfat milk for 1 h, and incubated with primary antibodies overnight at 4°C. After incubation with HRP‐conjugated secondary antibodies for 1 h at room temperature, signals were detected using an ECL substrate (Cat. No. UBI5044, YOBIBIO Biotechnology, Shanghai, China). Antibodies used were: Pan‐Kla antibody (Cat. No. P81825, Abmart, Shanghai, China; 1:1000), H3K9la antibody (Cat. No. PTM‐1419RM, PTM Bio, Hangzhou, China; 1:1000), H3K14la antibody (Cat. No. PTM‐1414RM, PTM Bio, Hangzhou, China; 1:1000), H3K18la antibody (Cat. No. PTM‐1427RM, PTM Bio, Hangzhou, China; 1:1000), H3K23la antibody (Cat. No. PTM‐1413RM, PTM Bio, Hangzhou, China; 1:1000), H3K27la antibody (Cat. No. PTM‐1428RM, PTM Bio, Hangzhou, China; 1:1000), H3K56la antibody (Cat. No. PTM‐1421RM, PTM Bio, Hangzhou, China; 1:1000), H3K27ac antibody (Cat. No. PTM‐116RM, PTM Bio, Hangzhou, China; 1:1000),Pan‐H3 antibody (Cat. No. ab1791, Abcam, Cambridge, UK; 1:5000), p16 antibody (Cat. No. 10883‐1‐AP, Proteintech, Wuhan, China; 1:1000), p21 antibody (Cat. No. 10355‐1‐AP, Proteintech, Wuhan, China; 1:1000), p53 antibody (Cat. No. 10442‐1‐AP, Proteintech, Wuhan, China; 1:5000), CARF antibody (Cat. No. 16615‐1‐AP, Proteintech, Wuhan, China; 1:500), PROS1 antibody (Cat. No. 16910‐1‐AP, Proteintech, Wuhan, China; 1:500), TYRO3 antibody (Cat. No. 28513‐1‐AP, Proteintech, Wuhan, China; 1:1000), AXL antibody (Cat. No. 13196‐1‐AP, Proteintech, Wuhan, China; 1:1000), MERTK antibody (Cat. No. 27900‐1‐AP, Proteintech, Wuhan, China; 1:1000), BAX antibody (Cat. No. 50599‐2‐Ig, Proteintech, Wuhan, China; 1:5000), GPX4 antibody (Cat. No. 67763‐1‐Ig, Proteintech, Wuhan, China; 1:1000), ACSL4 antibody (Cat. No. 22401‐1‐AP, Proteintech, Wuhan, China; 1:5000), p‐MLKL antibody (Cat. No. 82090‐2‐RR, Proteintech, Wuhan, China; 1:1000), MLKL antibody (Cat. No.  66675‐1‐Ig, Proteintech, Wuhan, China; 1:1000), FDX1 antibody (Cat. No. 12592‐1‐AP, Proteintech, Wuhan, China; 1:1000), DLAT antibody (Cat. No. 13426‐1‐AP, Proteintech, Wuhan, China; 1:1000), and Beta Actin antibody (Cat. No. 66009‐1‐Ig, Proteintech, Wuhan, China; 1:10000).

### 4.12 Quantitative Reverse Transcription Polymerase Chain Reaction (qRT‐PCR)

Total RNA was extracted using the RNeasy Kit (Cat. No. 74181, QIAGEN, Hilden, Germany) and quantified using a NanoDrop 2000 spectrophotometer. One microgram of total RNA was reverse‐transcribed using HiScript II Reverse Transcriptase (Cat. No. R323‐01, Vazyme, Nanjing, China). qPCR was performed using SYBR Green Master Mix (Cat. No. 4913914001, Roche, Basel, Switzerland) on an ABI 7500 Real‐Time PCR System. Primer sequences are listed in Table S1. Relative gene expression was calculated using the 2^−^ΔΔCt method with GAPDH as the internal control.

### 4.13 Immunofluorescence (IF)

Cells grown on coverslips or confocal dishes were fixed with 4% paraformaldehyde (Cat. No. P0099, Beyotime, Shanghai, China) for 15 min at room temperature, permeabilized with Triton X‐100 (Cat. No. T8787, Sigma–Aldrich, St. Louis, MO, USA) for 10 min, and blocked with BSA (Cat. No. A8020, Solarbio, Beijing, China) for 30 min. Samples were incubated overnight at 4°C with primary antibodies against p16(Cat. No. 80772‐1‐RR, Proteintech, Wuhan, China; 1:200), p21 (Cat. No. 10355‐1‐AP, Proteintech, Wuhan, China; 1:200) and Ki‐67 (Cat. No. R10018, ZenBio, Chengdu, China; 1:400). Alexa Fluor 488/555‐conjugated secondary antibodies (Cat. No. A11008/A21428, Invitrogen, Carlsbad, CA, USA; 1:500) were applied for 1 h at room temperature in the dark. Nuclei were counterstained with DAPI (Cat. No. C0065, Solarbio, Beijing, China) for 5 min. Coverslips were mounted using mounting medium (Cat. No. S2100, Solarbio, Beijing, China) and imaged with a confocal microscope (Olympus FV3000).

### 4.14 Lactate Quantification

Culture supernatants or cell lysates were collected, and lactate levels were measured using a lactate assay kit (Cat. No. BC2235, Solarbio, Beijing, China). Fifty microliters of supernatant was mixed with 150 µL of working solution containing the enzyme mix and chromogenic substrate, incubated at 37°C for 10 min in the dark, and then processed for absorbance measurement at 530 nm. Lactate concentrations were calculated from a standard curve.

### 4.15 Enzyme‐Linked Immunosorbent Assay (ELISA)

Secreted PROS1 in culture supernatants was quantified using a Human PROS1 ELISA kit (Cat. No. EH20057, Fine Biotech, Wuhan, China). Standards and samples were added to anti‐PROS1‐coated plates and incubated sequentially with a biotin‐conjugated detection antibody and HRP‐streptavidin. TMB substrate was used for color development, and optical density was measured at 450 nm. PROS1 concentrations were determined from a standard curve.

### 4.16 Flow Cytometry Cell Cycle Analysis

Cell cycle distribution was assessed using a cell cycle detection kit (Cat. No. CCS012, MultiSciences, Hangzhou, China) based on propidium iodide (PI) staining with RNase treatment. Approximately 1 × 10^6^ cells were harvested, washed with PBS, and incubated with 1 mL of DNA staining solution and 10 µL of permeabilization reagent for 30 min at room temperature in the dark. Samples were analyzed on a BD FACSCanto II flow cytometer with 488‐nm excitation, and cell cycle phase distributions were quantified using FlowJo software.

### 4.17 CUT&Tag

CUT&Tag was performed using a commercial CUT&Tag kit (Cat. No. TD903, Vazyme, Nanjing, China). Fresh cells (5 × 10^5^) were bound to ConA beads, incubated with primary antibodies overnight at 4°C, followed by incubation with a secondary antibody (anti‐rabbit IgG, 1:100) for 1 h at room temperature, pA/G‐Tn5 transposase assembly, and Mg^2^^+^‐activated tagmentation. DNA was then extracted, and sequencing libraries were prepared. Libraries were sequenced on an Illumina NovaSeq 6000 platform (PE150). Sequencing reads were aligned with Bowtie2, peaks were called using MACS2 (q < 0.05), and data were visualized with IGV.

For CUT&Tag‐qPCR validation of H3K27la enrichment at the PROS1 promoter, DNA was extracted using CUT&Tag Stop Buffer for qPCR (Cat. No. TD904‐C1, Vazyme, Nanjing, China) and subjected to qPCR analysis. Two independent primer pairs targeting the H3K27la peak region at the PROS1 promoter were designed based on CUT&Tag sequencing results. qPCR was performed using SYBR Green Master Mix (Roche, Cat. No. 4913914001) on an ABI 7500 Real‐Time PCR System. Data were normalized to spike‐in chromatin and presented as fold enrichment over IgG control.

### 4.18 Dual‐Luciferase Reporter Assay

The PROS1 promoter region (−2000 to +500 bp) was cloned into the pGL3‑Basic luciferase reporter vector (GeneCreate, Wuhan, China). Cells were co‑transfected with pGL3‑PROS1‑Luc and pRL‑CMV Renilla normalization plasmid using Lipofectamine 3000. After 24 h, cells were treated with 2‑DG (10 mM) (Cat. No. HY‐13966, MedChemExpress, Monmouth Junction, NJ, USA), Oxamate (20 mM) (Cat. No. HY‐W013032A, MedChemExpress, Monmouth Junction, NJ, USA), or combined with sodium lactate (10 mM) (Cat. No. L7022, Sigma–Aldrich, St. Louis, MO, USA) for 48 h. Firefly and Renilla luciferase activities were measured sequentially using the Dual‑Luciferase Reporter Assay Kit (Cat. No. JKR23008, GeneCreate, Wuhan, China). Firefly luciferase activity was normalized to Renilla luciferase activity, and data were expressed as fold change relative to control.

### 4.19 IP‐MS and Co‐IP

Cells were lysed in IP lysis buffer (Cat. No. 87787, Thermo Fisher Scientific, Waltham, MA, USA) and incubated overnight at 4°C with 5 µg of antibody under rotation, followed by incubation with Protein A/G magnetic beads (Cat. No. 88803, Thermo Fisher Scientific, Waltham, MA, USA) for 4 h at 4°C. After extensive washing, eluates were subjected either to SDS‐PAGE followed by silver staining and mass spectrometry or to Western blotting. IP‐MS analysis was performed by GeneChem (Shanghai, China). Briefly, immunoprecipitated complexes were digested with trypsin and analyzed by LC‐MS/MS on an Orbitrap Fusion Lumos instrument. Data were processed using MaxQuant against the UniProt human database, and peptides were filtered at a false discovery rate (FDR) of <1% with at least two unique peptides.

### 4.20 Molecular Docking

Protein‐protein docking between TYRO3 and CARF was performed using the HDOCK server (http://hdock.phys.hust.edu.cn/). The optimal complex conformations were selected based on docking scores and energy rankings. 3D structures were visualized using PyMOL (v2.5, Schrödinger, LLC) to analyze binding interfaces, hydrogen bonding, and hydrophobic interactions.

### 4.21 CHX Treatment

Cells were seeded in 6‐well plates and treated with cycloheximide (CHX) (Cat. No. HY‐12320, MedChemExpress, Monmouth Junction, NJ, USA) at 100 µg/mL for 0, 1, 3, or 5 h. Cell lysates were collected, and residual target protein levels were assessed by Western blotting to estimate protein half‐life.

### 4.22 MG132 Treatment

Cells were treated with MG132 (Cat. No. HY‐13259, MedChemExpress, Monmouth Junction, NJ, USA) at 10 µmol/L for 5 h. In CHX chase experiments, CHX and MG132 were added simultaneously to inhibit proteasomal degradation. Cell lysates were analyzed by Western blotting.

### 4.23 Phos‐Tag SDS‐PAGE

Phos‐tag SDS‐PAGE was performed using Phosbind Acrylamide (Cat. No. F4002, APExBIO, Houston, TX, USA) at a final concentration of 50 µmol/L together with 100 µmol/L MnCl_2_ in the separating gel; the stacking gel contained no Phos‐tag reagent. Proteins were resolved at 80 V until the dye front entered the separating gel and then at 150 V for 90–120 min. After electrophoresis, gels were incubated in transfer buffer containing 10 mmol/L EDTA for 20 min to chelate Mn^2^^+^, followed by incubation in EDTA‐free transfer buffer for 10 min. Proteins were then transferred onto PVDF membranes and analyzed by routine Western blotting.

### 4.24 In Vivo Xenograft Tumor Model

Four‐ to six‐week‐old female BALB/c nude mice (weighing 18–22 g) were obtained from Shanghai Kingbio Biosciences Inc. (Shanghai, China) and housed under specific pathogen‐free conditions with a 12‐h light/dark cycle and ad libitum access to food and water. All animal experiments were conducted at the animal research facility of Shanghai Kingbio Biosciences Inc. under the approval and oversight of its Institutional Animal Care and Use Committee (IACUC). IOMM‐LeeR stable cell lines carrying the indicated genetic modifications were inoculated subcutaneously at 2 × 10^6^ cells per mouse. Tumor volume was calculated using the formula length × width^2^ × 0.5. Once tumors were established, mice were randomized into the following groups (using a random number table; n = 6 mice per group): NC, NC + IR, PROS1‐KD + IR, PROS1‐KD + CARF‐KD + IR, PROS1‐KD + CARF‐KD + WT‐OE + IR, and PROS1‐KD + CARF‐KD + Y8F‐OE + IR. Local single‐dose irradiation (16 Gy) was administered on day 6, with the remainder of the body shielded by lead. Tumor size was measured every 3 days. Mice were euthanized on day 27, and tumors were excised for subsequent analyses. Humane endpoints were strictly observed throughout the study; no animals were euthanized prematurely due to tumor burden or distress, as all tumors remained below the predefined humane limit (2,000 mm^3^) and no severe adverse events were observed.

### 4.25 Clinical Specimen Collection

Formalin‐fixed, paraffin‐embedded (FFPE) meningioma specimens were retrospectively collected from the institutional pathology archive of Ruijin Hospital, Shanghai Jiao Tong University School of Medicine, where all patients underwent surgical resection for meningioma. The specimens comprising two independent cohorts: (i) radiotherapy‐naive primary meningiomas from patients who underwent upfront surgical resection without prior radiotherapy, and (ii) post‐irradiation recurrent meningiomas from patients who experienced tumor recurrence after photon‐based radiotherapy. The exact sample size for each group is provided in the corresponding figure legends. All specimens were independently reviewed by two neuropathologists to confirm the diagnosis. Inclusion criteria included adequate FFPE tissue availability and complete clinicopathological information relevant to the present study. Exclusion criteria included insufficient tissue quality, ambiguous pathological diagnosis, or incomplete clinical information.

### 4.26 Ethics Statement

All experimental protocols involving human tissue specimens were approved by the Ethics Committee of Ruijin Hospital, Shanghai Jiao Tong University School of Medicine (Approval No. KY‐2021‐787, issued on November 28, 2021). This approval covered the acquisition of patient‐derived meningioma specimens as well as the derivation, and experimental use of the MN2202 and MN2208 cell lines. Written informed consent was obtained from all patients prior to sample collection. All human specimen‐related procedures were conducted in accordance with the principles of the Declaration of Helsinki and the relevant guidelines and regulations of Ruijin Hospital, Shanghai Jiao Tong University School of Medicine.

All animal experiments were approved by the Institutional Animal Care and Use Committee (IACUC) of Shanghai Kingbio Biosciences Inc. (Approval No. 2026‐002, issued on March 20, 2026). All animal procedures were conducted at the animal research facility of Shanghai Kingbio Biosciences Inc. under the oversight of the approving institution and in strict accordance with the Regulations for the Administration of Laboratory Animals promulgated by the National Science and Technology Commission of China and the guidelines of the IACUC of Shanghai Kingbio Biosciences Inc.

### 4.27 Hematoxylin and Eosin (H&E) Staining

Tumor tissues were fixed, paraffin‐embedded, and sectioned at 4 µm thickness. Sections were deparaffinized, rehydrated, stained with hematoxylin and eosin according to standard histological procedures, and mounted for microscopic examination.

### 4.28 Immunohistochemistry

Paraffin‐embedded sections were deparaffinized, rehydrated, and subjected to antigen retrieval in sodium citrate buffer (pH 6.0) by microwave heating for 15 min. Endogenous peroxidase activity was blocked with 3% H_2_O_2_ for 10 min. Sections were incubated overnight at 4°C with primary antibodies, HRP‐conjugated secondary antibodies were applied for 30 min at room temperature, followed by DAB chromogenic development and hematoxylin counterstaining.

### 4.29 Statistical Analysis

Data were pre‐processed and analyzed using GraphPad Prism 10.0 (GraphPad Software, San Diego, CA, USA). All continuous variables were first assessed for normality using the Shapiro‐Wilk test and for homogeneity of variance using Levene's test, where applicable. Data meeting the assumptions of normal distribution are presented as mean ± standard deviation (SD). For comparisons between two independent groups, an unpaired two‐tailed Student's t‐test was used. For comparisons involving more than two groups at a single time point, one‐way analysis of variance (ANOVA) followed by Tukey's multiple‐comparisons test was performed. For longitudinal datasets involving multiple time points, including CCK‐8 assays and xenograft tumor‐growth curves, two‐way ANOVA was used to evaluate the effects of treatment, time, and their interaction. The exact statistical test used for each dataset is specified in the corresponding figure legend, the meaning of n is also defined in each figure legend and refers. A two‐sided *p* value < 0.05 was considered statistically significant.

## Author Contributions

Conceptualization: Z.W., J.C., L.B., B.W. Methodology: Z.W., Q.Z., K.L., X.L., H.Z., Y.C., Y.M., Y.L., A.X., Y.S., J.C., L.B., B.W. Software: Z.W., Q.Z., K.L., X.L., H.Z., Y.C., Y.M., Y.L., A.X., Y.S., J.C., L.B., B.W. Validation: Z.W., Q.Z., K.L., X.L., H.Z., Y.C., Y.M., Y.L., A.X., Y.S., J.C., L.B., B.W. Formal analysis: Z.W., Q.Z., X.L., K.L., H.Z., Y.C., Y.M., Y.L., A.X., Y.S., J.C., L.B., B.W. Investigation: Z.W., Q.Z., K.L., X.L., Y.S., J.C., L.B., B.W. Resources: Z.W., Q.Z., K.L., X.L., J.C., L.B., B.W. Data curation: Z.W., Q.Z., X.L., K.L., H.Z., J.C., L.B., B.W. Writing – original draft: Z.W., Q.Z., K.L., X.L., B.W. Writing – review & editing: Z.W., Q.Z., K.L., X.L., J.C., L.B., B.W. Visualization: Z.W., Q.Z., K.L., X.L., H.Z., Y.C., Y.M., Y.L., A.X., Y.S., J.C., L.B., B.W. Supervision: Z.W., Q.Z., K.L., X.L., J.C., L.B., B.W. Project administration: Z.W., Q.Z., X.L., K.L., H.Z., Y.C., J.C., L.B., B.W. All authors read and approved the final manuscript.

## Conflicts of Interest

The authors declare no conflicts of interest.

## AI Disclosure Statement

The authors confirm that no generative artificial intelligence, large language model, or other AI‐based content‐generation tools were used at any stage of manuscript preparation, including writing, data analysis, result interpretation, figure generation, or revision. All scientific content was performed entirely by the human authors.

## Supporting information




**Supporting File 1**: advs77818‐sup‐0001‐Table1.xlsx.

**Supporting File 2**: advs77818‐sup‐0002‐SuppMat.docx.

## Data Availability

The data that support the findings of this study are openly available, raw sequencing data (SRA: PRJNA1508292), proteomics data (iProX: PXD082306), and metabolomics data (MetaboLights: MTBLS15274) have been deposited in public repositories.
