# Supplementary material for: Lactylation‐Driven PROS1‐TYRO3‐CARF Signaling Promotes Therapy‐Induced Senescence Escape and Radioresistance in Meningioma
Source: Adv Sci (Weinh). 2026 Sep 16:e77818. Online ahead of print. doi: 10.1002/advs.77818 (PMC13583086; doi:10.1002/advs.77818)
Supplement: Supplementary file 1 [file ADVS-9999-e77818-s002.docx]

**Figure S1. Establishment and characterization of radioresistant meningioma cell lines.**

**(A)** Immunofluorescence of Vimentin and EMA in two patient-derived benign meningioma immortalized cell lines (MN2202 and MN2208), confirming meningioma origin. Scale bar: 100 μm.

**(B)** Representative colony formation images of parental and radioresistant IOMM Lee and MN2202 cells following single dose irradiation at the indicated doses (0, 2, 4, 6, and 8 Gy).

**(C)** Survival curves of IOMM-Lee and IOMM-LeeR cells derived from colony formation assays. (n = 3). Two-way ANOVA test was applied.

**(D)** Survival curves of MN2202 and MN2202R cells derived from colony formation assays. (n = 3). Two-way ANOVA test was applied.

**(E)** Immunofluorescence staining of γH2AX foci (green) in parental and radioresistant cells of both lines at 2 h post 8 Gy irradiation. DAPI (blue) was used for nuclear counterstaining. Scale bar: 50 μm (n = 3).

“n” means the number of individual experiments. Data are presented as mean ± SD. *P < 0.05; **P < 0.01; ***P < 0.001; ns, not significant.

**Figure S2. Cell cycle‑related phenotypes of radioresistant cells and validation of SASP‑mediated clonogenic enhancement via conditioned medium.**

**(A)** Representative flow cytometric cell-cycle histograms of groups in Figure 1F.

**(B)** Immunofluorescence of Ki67 (red) at pre-IR, D5, and D15. Scale bar: 100 μm.

**(C)** Quantification of Ki67 fluorescence intensity (n = 3). Unpaired two-tailed Student’s t-test was applied.

**(D)** qPCR analysis of cell cycle‑related genes (CCNA2, CCNB1, and PCNA) in IOMM‑Lee and IOMM‑LeeR cells at 0, 5, and 15 days post‑8 Gy irradiation (n = 3). One-way ANOVA followed by Tukey’s multiple-comparisons test was applied.

**(E)** Colony formation of IOMM‑Lee and MN2202 cells cultured in conditioned media from irradiated parental cells (0, 2, 4, 6, 8 Gy).

**(F)** Quantification of colony formation from **(E)** (n = 3). One-way ANOVA followed by Tukey’s multiple-comparisons test was applied.

“n” means the number of individual experiments. Data are presented as mean ± SD. *P < 0.05; **P < 0.01; ***P < 0.001; ns, not significant.

**Figure S3. Integrated metabolomic profiling of radioresistant meningioma cells and validation of glycolytic phenotype.**

**(A)** Volcano plot of differentially abundant metabolites between IOMM‑Lee and IOMM‑LeeR cells. Super Class classification of differential metabolites showing the relative abundance of each metabolite class in IOMM‑Lee vs. IOMM‑LeeR cells. n = 6 biological replicates per group.

**(B)** KEGG pathway enrichment analysis of differential metabolites (Top 20). The glycolysis/gluconeogenesis pathway is highlighted in red.

**(C)** DAScore_Plot showing the pathway impact score versus -log10(P‑value) for each enriched KEGG pathway. The glycolysis/gluconeogenesis pathway is highlighted in red.

**(D)** KEGG glycolytic pathway map with differential metabolites mapped onto the pathway. Red boxes indicate metabolites significantly increased in IOMM‑LeeR cells; blue boxes indicate decreased metabolites.

**(E)** Changes in culture supernatant pH of MN2202 and MN2202R cells at 0, 24, and 48 h (n = 3). Unpaired two-tailed Student’s t-test was applied.

**(F)** Lactate levels in culture media of MN2202 and MN2202R cells at 0, 24, and 48 h (n = 3). Unpaired two-tailed Student’s t-test was applied.

**(G)** Representative colony formation assay of parental IOMM‑Lee and MN2202 cells treated with 2‑DG (1 mM) or Oxamate (5 mM) without irradiation (n = 3). One-way ANOVA followed by Tukey’s multiple-comparisons test was applied.

“n” means the number of individual experiments. Data are presented as mean ± SD. *P < 0.05; **P < 0.01; ***P < 0.001; ns, not significant.

**Figure S4. Validation of senescence escape, lactylation, PROS1 transcriptional activation in the MN2202 radioresistant model, and Functional validation of recombinant PROS1 in benign meningioma cell lines.**

**(A)** Western blot analysis of senescence markers p16, p21, and p53 in MN2202 and MN2202R cells at D0, D5, D10, and D15 post‑8 Gy irradiation.

**(B)** Western blotting of global lactylation (Pan‑Kla) in MN2202 and MN2202R cells.

**(C)** Western blot of specific histone H3 lysine lactylation sites (H3K9la, H3K14la, H3K18la, H3K23la, H3K27la, H3K56la) in MN2202 and MN2202R cells.

**(D)** qPCR analysis of PROS1 mRNA levels in MN2202 and MN2202R cells treated with 2‑DG (10 mM), Oxamate (20 mM), or combined with exogenous sodium lactate (10 mM) (n = 3). One-way ANOVA followed by Tukey’s multiple-comparisons test was applied.

**(E)** Dual‑luciferase reporter assay of PROS1 promoter activity in MN2202 and MN2202R cells treated as in (D). Firefly luciferase activity was normalized to Renilla luciferase (n = 3). One-way ANOVA followed by Tukey’s multiple-comparisons test was applied.

**(F)** Representative colony formation images of MN2208 cells treated with increasing concentrations of recombinant PROS1 (0, 30, 60, 90 nM) after 6 Gy irradiation.

**(G)** Quantification of colony formation from (Fig3 O, MN2202) (n = 3). One-way ANOVA followed by Tukey’s multiple-comparisons test was applied.

**(H)** CCK‑8 cell viability assay of MN2202 cells treated with recombinant PROS1 (0, 30, 60, 90 nM) after 6 Gy irradiation (n = 3). Two-way ANOVA test was applied.

**(I)** Western blot analysis of p16, p21, and p53 in MN2202 cells treated with increasing concentrations of recombinant PROS1 (0, 30, 60, 90 nM) after 6 Gy irradiation.

**(J)** Quantification of colony formation from (F) (n = 3). One-way ANOVA followed by Tukey’s multiple-comparisons test was applied.

**(K)** CCK‑8 cell viability assay of MN2208 cells treated with recombinant PROS1 (0, 30, 60, 90 nM) after 6 Gy irradiation (n = 3). Two-way ANOVA test was applied.

**(L)** Western blot analysis of senescence markers p16, p21, and p53 in MN2208 cells treated with increasing concentrations of recombinant PROS1 (0, 30, 60, 90 nM) after 6 Gy irradiation.

“n” means the number of individual experiments. Data are presented as mean ± SD. *P < 0.05; **P < 0.01; ***P < 0.001; ns, not significant.

**Figure S5. PROS1-TYRO3 signaling in MN2202 and MN2208 cells.**

**(A)** Western blot analysis of representative markers associated with ferroptosis (GPX4, ACSL4), apoptosis (BAX), necroptosis (p-MLKL, MLKL), and cuproptosis (FDX1, DLAT) in control and PROS1-KD cells.

**(B)** Western blot of p16, p21, and p53 in MN2202 and MN2208 with NC, PROS1-OE, PROS1-OE + TYRO3-KD, PROS1-OE + AXL-KD, PROS1-OE + MERTK-KD at D15 post-6 Gy.

**(C)** Western blot of p16, p21, and p53 in MN2202 cells with NC, PROS1‑OE, UNC9426 (TYRO3 inhibitor, 200 nM). PROS1‑OE + UNC9426.

**(D)** Immunofluorescence of Ki67 (red) in IOMM-Lee with NC, PROS1-OE, TYRO3-KD, and PROS1-OE + TYRO3-KD at D15 post-6 Gy. Scale bar: 100 μm.

**(E)** Quantification of Ki67 fluorescence intensity (n = 3). One-way ANOVA followed by Tukey’s multiple-comparisons test was applied.

**(F)** qPCR analysis of cell cycle‑related genes (CCNA2, CCNB1, and PCNA) in NC, PROS1-OE, TYRO3-KD, and PROS1-OE + TYRO3-KD groups (n = 3). One-way ANOVA followed by Tukey’s multiple-comparisons test was applied.

“n” means the number of individual experiments. Data are presented as mean ± SD. *P < 0.05; **P < 0.01; ***P < 0.001; ns, not significant.

**Figure S6. Functional validation of PROS1-TYRO3 axis in benign meningioma cell lines.**

**(A)** Representative flow cytometric cell-cycle histograms of NC, PROS1-OE, TYRO3-KD, and PROS1-OE + TYRO3-KD groups.

**(B-C)** Representative colony formation and quantification for NC, PROS1-OE, TYRO3-KD, and PROS1-OE + TYRO3-KD groups in the MN2202 cell line after 6 Gy irradiation (n = 3). One-way ANOVA followed by Tukey’s multiple-comparisons test was applied.

**(D)** CCK-8 cell viability assay in the MN2202 cell line for the indicated groups (n = 3). Two-way ANOVA test was applied.

**(E-F)** Representative colony formation and quantification for PROS1-OE, TYRO3-KD, and PROS1-OE + TYRO3-KD groups in the MN2208 cell line after 6 Gy irradiation (n = 3). One-way ANOVA followed by Tukey’s multiple-comparisons test was applied.

**(G)** CCK-8 cell viability assay in the MN2208 cell line for the indicated groups (n = 3). Two-way ANOVA test was applied.

“n” means the number of individual experiments. Data are presented as mean ± SD. *P < 0.05; **P < 0.01; ***P < 0.001; ns, not significant.

**Figure S7. Validation of CARF-TYRO3 interaction and CARF degradation mechanism in benign meningioma cell lines.**

**(A)** Co-IP of endogenous TYRO3-CARF interaction in MN2202 and MN2208.

**(B)** Quantitative analysis of TYRO3-CARF co-localization in IOMM-Lee.

**(C)** Co‑IP of CARF with p16 and p53 in IOMM‑Lee cells.

**(D)** CHX chase assay of p16 and p53 protein stability in control and CARF‑KD IOMM‑Lee cells. Cells were treated with CHX for 0, 1, 2, and 3 h.

**(E)** CHX chase assay of p16 and p53 protein stability in control and PROS1-OE IOMM‑Lee cells. Cells were treated with CHX for 0, 1, 3, and 5 h.

**(F)** Ubiquitination assay of CARF in IOMM‑Lee and MN2202 cells with or without PROS1-OE. Cells were treated with MG132.

**(G, H)** Western blot of p16, p21, and p53 in MN2202 (G) and MN2208 (H) with NC, CARF-KD, CARF-KD + WT-OE, and CARF-KD + Y8F-OE groups post-6 Gy.

**(I)** CHX chase of exogenous WT or Y8F CARF in MN2202 and MN2208.

**(J)** Immunofluorescence of p21 (green) and p16 (red) in IOMM-Lee with the four groups at D15 post-8 Gy. Scale bar: 50 μm.

**Figure S8. Functional validation of CARF phosphorylation site in benign meningioma cell lines.**

**(A)** Representative flow cytometric cell-cycle histograms of NC, CARF-KD, CARF-KD + WT-OE, and CARF-KD + Y8F-OE groups

**(B-C)** Representative colony formation and quantification for NC, CARF-KD, CARF-KD + WT-OE, and CARF-KD + Y8F-OE groups in the MN2202 cell line after 6 Gy irradiation (n = 3). One-way ANOVA followed by Tukey’s multiple-comparisons test was applied.

**(D)** CCK-8 cell viability assay in the MN2202 cell line for the indicated groups (n = 3). Two-way ANOVA test was applied.

**(E-F)** Representative colony formation and quantification for NC, CARF-KD, CARF-KD + WT-OE, and CARF-KD + Y8F-OE groups in the MN2208 cell line after 6 Gy irradiation (n = 3). One-way ANOVA followed by Tukey’s multiple-comparisons test was applied.

**(G)** CCK-8 cell viability assay in the MN2208 cell line for the indicated groups (n = 3). Two-way ANOVA test was applied.

“n” means the number of individual experiments. Data are presented as mean ± SD. *P < 0.05; **P < 0.01; ***P < 0.001; ns, not significant.

**Figure S9.  Quantification of immunohistochemical staining in clinical specimens and subcutaneous xenograft tumors.**

**(A)** Quantification of IHC staining in patient samples shown in Figure 7A (N = 6/group). Unpaired two-tailed Student’s t-test was applied.

**(B)** Representative H&E staining, and IHC images of pan‑Kla, H3K27la, PROS1, TYRO3, and pTYRO3 in tumor sections from the identical treatment groups as in Figure 7F. Scale bar: 100 μm

**(C)** Quantification of IHC staining in subcutaneous xenograft tumor tissues shown in Figure 7G and Figure S9B (N = 6/group). One-way ANOVA followed by Tukey’s multiple-comparisons test was applied.

“N” means the number of subjects. Data are presented as mean ± SD. *P < 0.05; **P < 0.01; ***P < 0.001; ns, not significant.
